# Computing the Local Field Potential (LFP) from Integrate-and-Fire Network Models

**DOI:** 10.1371/journal.pcbi.1004584

**Published:** 2015-12-14

**Authors:** Alberto Mazzoni, Henrik Lindén, Hermann Cuntz, Anders Lansner, Stefano Panzeri, Gaute T. Einevoll

**Affiliations:** 1 The Biorobotics Institute, Scuola Superiore Sant’Anna, Pontedera, Pisa, Italy; 2 Neural Computation Laboratory, Center for Neuroscience and Cognitive Systems @UniTn, Istituto Italiano di Tecnologia, Rovereto, Italy; 3 Department of Neuroscience and Pharmacology, University of Copenhagen, Copenhagen, Denmark; 4 Department of Computational Biology, School of Computer Science and Communication, Royal Institute of Technology–KTH, Stockholm, Sweden; 5 Ernst Strüngmann Institute (ESI) for Neuroscience in Cooperation with Max Planck Society, Frankfurt/Main, Germany; 6 Institute of Clinical Neuroanatomy, Goethe University Frankfurt, Frankfurt/Main, Germany; 7 Frankfurt Institute for Advanced Studies (FIAS), Frankfurt/Main, Germany; 8 Department of Mathematical Sciences and Technology, Norwegian University of Life Sciences, Ås, Norway; 9 Department of Physics, University of Oslo, Oslo, Norway; University College London, UNITED KINGDOM

## Abstract

Leaky integrate-and-fire (LIF) network models are commonly used to study how the spiking dynamics of neural networks changes with stimuli, tasks or dynamic network states. However, neurophysiological studies *in vivo* often rather measure the mass activity of neuronal microcircuits with the local field potential (LFP). Given that LFPs are generated by spatially separated currents across the neuronal membrane, they cannot be computed directly from quantities defined in models of point-like LIF neurons. Here, we explore the best approximation for predicting the LFP based on standard output from point-neuron LIF networks. To search for this best “LFP proxy”, we compared LFP predictions from candidate proxies based on LIF network output (e.g, firing rates, membrane potentials, synaptic currents) with “ground-truth” LFP obtained when the LIF network synaptic input currents were injected into an analogous three-dimensional (3D) network model of multi-compartmental neurons with realistic morphology, spatial distributions of somata and synapses. We found that a specific fixed linear combination of the LIF synaptic currents provided an accurate LFP proxy, accounting for most of the variance of the LFP time course observed in the 3D network for all recording locations. This proxy performed well over a broad set of conditions, including substantial variations of the neuronal morphologies. Our results provide a simple formula for estimating the time course of the LFP from LIF network simulations in cases where a single pyramidal population dominates the LFP generation, and thereby facilitate quantitative comparison between computational models and experimental LFP recordings *in vivo*.

## Author Summary

Leaky integrate-and-fire (LIF) networks are often used to model neural network activity. The spike trains they produce, however, cannot be directly compared to the local field potentials (LFPs) that are measured by low-pass filtering the potential recorded from extracellular electrodes. This is because LFPs are generated by neurons with spatial extensions, while LIF networks typically consist of point neurons. In order to still be able to approximately predict LFPs from LIF network simulations, we here explore simple proxies for computing LFPs based on standard output from LIF network simulations. Predictions from the various LFP proxies were compared with “ground-truth” LFPs computed by means of well-established volume conduction theory where synaptic currents corresponding to the LIF network simulation were injected into populations of multi-compartmental neurons with realistic morphologies. We found that a simple weighted sum of the LIF synaptic currents with a single universally applicable set of weights excellently capture the time course of the LFP signal when the LFP predominantly is generated by a single population of pyramidal cells. Our study therefore provides a simple formula by which the LFP signal can be estimated directly from the LIF network activity, providing a missing quantitative link between simple neural models and LFP measures *in vivo*.

## Introduction

Models of recurrently connected networks of leaky integrate-and-fire (LIF) neurons are well established tools for studying brain function [1,2]. The equations describing the single LIF neuron are simple and can be easily adapted to generate complex dynamics [3,4]. Despite their simplicity, LIF network models have proved able to describe a wide spectrum of different cortical dynamics and cortical functions, from the emergence of up and down states [5–7], working memory [8–10], attention [11,12], decision making [13], rhythmogenesis [14], and sensory information coding [11,15,16]. In some cases it is possible to describe the dynamics of LIF networks analytically [17,18], thus providing deeper insights into how spiking neuronal networks may implement the basic cerebral computational mechanisms [19].

Models can only be properly tested against experimental evidence when they can predict empirical measures quantitatively. Local cortical activity is often recorded *in vivo* or *in vitro* using the local field potential (LFP), a measure obtained by low-pass filtering (below a few hundred hertz) the electrical potential recorded from extracellular electrodes. The LFP signal reflects mass neural activity arising within a few hundred micrometers or more from the recording electrode [20–25]. This spatial scale is highly relevant for LIF network models, which typically aim to describe the activity of thousands or tens of thousands of cells. The recording of LFPs has a prominent role in systems neuroscience, and such recordings have been used extensively to investigate cortical network mechanisms involved in sensory processing [26], motor planning [27], and higher cognitive processes [28].

LFP is generated by transmembrane currents in the neurons in the vicinity of the recording electrode [23] and depends on morphological features of the contributing cells, the positioning of synapses, as well as the correlation level of synaptic inputs [20,21,29,30]. Under reasonable assumptions about the extracellular milieu the cellular LFP contributions can be computed as a weighted sum of the transmembrane currents in multi-compartment neuron models [31–34]. This allows for detailed numerical investigations of spatial, as well as spectral features of the LFP signals [35]. In particular, such simulations of large populations of morphologically detailed neurons have provided insight into how the neuronal activity at the population level influences the spatial reach and laminar variation of the LFP signal *in vivo* [20,21,33,34,36] the relative importance of active and passive currents [37], and the population LFP signal measured from cortical slices in microelectrode arrays (MEAs) [38,39].

However, it has been unclear how best to use LIF networks to model and provide understanding of LFP recordings. This is because extracellular potentials arise in biological tissue due to a spatial separation of inward (sinks) and outward (sources) transmembrane currents of the neurons, and neuron models used to compute an LFP signal must thus have a minimum of two spatially separated compartments in order to generate a potential [32]. In LIF models, however, a single compartment is typically used as an approximation of the entire neuron, including the spatially extended dendritic structure, and individual cells within a population are not assigned to a specific spatial position.

One possible way to compute LFPs from LIF network is to project the spike times generated by the LIF network under consideration onto morphologically detailed 3D neuron models and then compute the field that the currents flowing through these 3D networks generate. However, this approach would require the modeler to set up a cumbersome and computationally expensive network model based on multi-compartment model neuron. As a much simpler alternative, we here instead search for a general and easy-to-use proxy to predict the time course of the LFP based on variables available directly from the LIF network simulations. Here we investigate and evaluate different strategies to compute an LFP proxy directly from the output of standard LIF network simulations without the use of multi-compartment neuronal morphologies.

Our approach is as follows: we first simulate an LIF point-neuron network model and record the output spiking activity, membrane potentials, and synaptic currents. Next, we compute a realistic ground-truth estimate of the LFP that the same LIF network activity would generate. We do this by injecting distributed synaptic currents corresponding to the stored LIF synaptic events, onto a population of multi-compartment neurons with realistic distributions of dendrites and synapses (we call this population the “3D network”). We then compare this simulated ground-truth LFP signal to a number of LFP proxies computed directly from measures of activity of the point-neuron LIF network. These proxies include those previously proposed in the literature (e.g., the average firing rate [11,14,40], the average membrane potential [24,41–44], the sum of synaptic currents [7,45], and the sum of absolute values of synaptic currents [15]), as well as others proposed here. By separating the spiking dynamics generated by the LIF network from the LFP generated by the 3D network, we are also able to investigate how different assumptions regarding cell morphology, synaptic distributions and recording positions influence the accuracy of the different LFP proxies.

We find that a simple linear combination of excitatory (AMPA) and inhibitory (GABA) synaptic currents extracted from the point-neuron LIF network provides a proxy for the LFP that closely matches the temporal features of the signals resulting from the morphologically realistic LFP model generated by the 3D network. Even with a small set of fixed parameters this LFP proxy is able to account for the LFP signal with a high degree of precision under most investigated conditions.

## Results

### A morphological model for a cortical LIF network

Our goal was to understand how to compute a simple yet accurate approximation (denoted as “proxy” in the following) of the LFP that would be generated by the time series of synaptic activity of an LIF network if its neurons had a realistic spatial structure and arrangement. We therefore first simulated an LIF network (known to reproduce several features of cortical dynamics). Next, we injected the synaptic activity it generated into a synthetic three-dimensional network model (3D network) of a layer of a cortical column that employed multi-compartmental neurons with realistic morphology, spatial distributions of somata and synapses, and computed the extracellular potentials generated by this synaptic activity.

We selected an LIF network (adapted from [14] and refined in [15,16,46,47]) that has been shown to reproduce a number of important features of the dynamics of visual primary cortical neural population recorded *in vivo* during naturalistic sensory stimulation, including a realistic spectrum of cortical dynamics and of its modulation with the visual stimuli, including low-frequency (1–12 Hz) and gamma (50–100 Hz) oscillations [15,46]. Moreover, when using a simple proxy (which is demonstrated below to perform well) to compute an LFP from synaptic currents, this LIF network reproduced quantitatively several important properties of recorded extracellular potentials, including LFP power spectra and spectral information content [15], and cross-frequency and spike-field relationships [16,46]. Thus, the LIF network seemed to generate a sufficiently realistic dynamics to provide synaptic input for the generation of biologically plausible LFPs in the 3D network.

**Fig 1:**
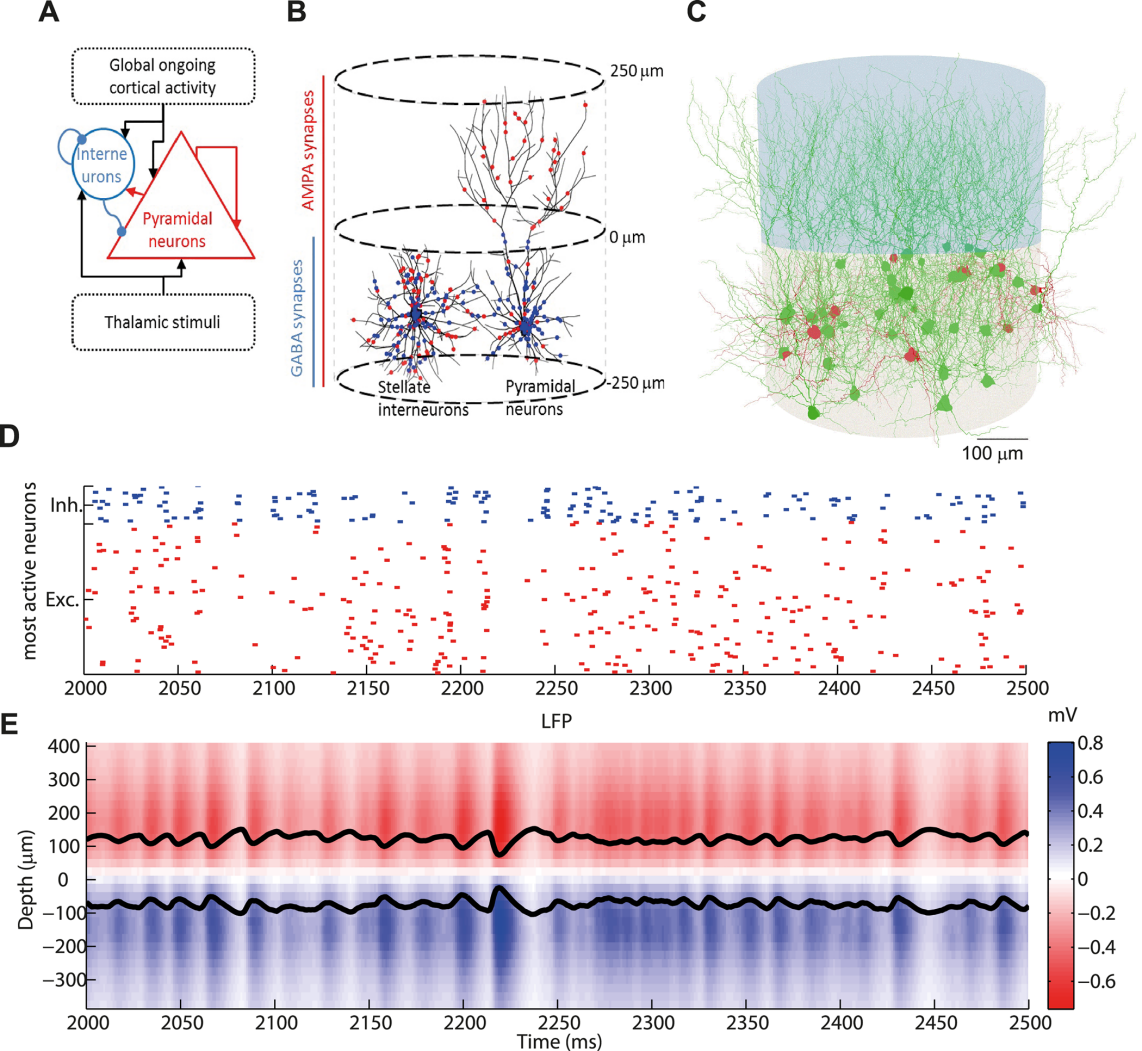
LIF network and 3D morphological network. (A) Sketch of the leaky integrate-and-fire (LIF) network. A population of 1000 interneurons with GABA synapses (blue) and a population of 4000 pyramidal neurons with AMPA synapses (red) receive recurrent inputs (random connectivity with 20% probability) and two kinds of external inputs: global ongoing cortical activity (Ornstein-Uhlenbeck process) and a regular thalamic stimulation. (B) Sketch of the morphological 3D network made of two stacked cylinders with 250 μm radius and 250 μm height. A representative interneuron and pyramidal cell are depicted. Interneuron dendrites remain in the lower cylinder while the pyramidal neuron dendrites reach out to the upper cylinder. Dendrites in the lower cylinder receive both AMPA and GABA synapses while dendrites in the upper cylinder receive only AMPA synapses. (C) Graphical rendering of a subset of the 3D network composed of 10 interneurons and 40 pyramidal neurons. (D) Raster plot of the spiking activity of the 10 interneurons (blue, top) and the 40 pyramidal neurons (red, bottom) with the highest spiking activity in the LIF network, for a thalamic stimulation of 1.5 spikes/ms. (E) Depth-resolved LFP signal as simulated by injecting the spikes generated by the whole network during thalamic stimulation of 1.5 spikes/ms into the 3D network. Black lines show LFP for 100 and -100 μm depth.

The LIF network model (Fig 1A) was composed of 4000 excitatory and 1000 inhibitory LIF neurons that were randomly connected with a pair-wise connection probability of 0.2 (for further details see Methods). The LIF network received two kinds of external inputs: a “thalamic” synaptic input thought to carry the information about the external stimuli and a stimulus-unrelated input representing slow ongoing fluctuations of activity. Synaptic dynamics and parameters are reported in Tables 1 and 2, and further details can be found in the Methods. Importantly, as is the case for most LIF network models to date, our LIF network did not have any spatial structure: the individual neurons were not assigned to a specific spatial position and consequently the connectivity had a random and sparse structure.

**Table 1:** Summary of leaky integrate-and-fire (LIF) network model.

**A–Leaky integrate and fire model summary**			
**Populations**	excitatory, inhibitory		
**Topology**	-		
**Connectivity**	Random and sparse		
**Neuron model**	Leaky integrate and fire, fixed threshold, fixed refractory time		
**Synapse model**	Difference of exponential functions defined by rise and decay time.Current-based synapses.		
**Plasticity**	-		
**Input**	Sum of independent Poisson processes with same time-varying rate for all neurons		
**Measurements**	For each population: firing rate, GABA and AMPA currents, membrane potential		
**B–Populations**			
**Type**	**Elements**		
Interneurons (GABA synapses)	LIF neurons		
Pyramidal neurons (AMPA synapses)	LIF neurons		
**C–Connectivity**			
**Name**	**Source**	**Target**	**Pattern**
AMPA_cor-Pyr	Pyramidal	Pyramidal	dir. conn. p_dc_ weight: J_AMPA_cor/pyr_
AMPA_cor-Inter	Pyramidal	Interneuron	dir. conn. p_dc_ weight: J_AMPA_cor/inter_
GABA-Pyr	Interneuron	Pyramidal	dir. conn. p_dc_ weight: J_GABA/pyr_
GABA-Inter	Interneuron	Interneuron	dir. conn. p_dc_ weight: J_GABA/inter_
AMPA_th-Pyr	External	Pyramidal	Uniform, J_AMPA_th/pyr_
AMPA_th-Inter	External	Interneuron	Uniform, J_GABA_th/pyr_
**D- Neuron**			
**Type**	**Leaky integrate and fire**		
**Description**	Subthreshold dynamics: $\tau_{m}{\dot{V}}_{m}\left(t\right)=-V_{m}\left(t\right)+\sum PSC_{syn}\left(t\right)$		
	If *V*_*m*_ *>V*_*thr*_ & t>t*+τ_*refractory*_		
	{t* = t; spike emitted with time stamp t*; V_m_ = V_reset_}		
**E–Synapse**			
**Type**	**Current synapse**		
**Description**	$\tau_{dsyn}\dot{PSC}\left(t\right)=-PSC\left(t\right)+x\left(t\right)$		
	$\tau_{rsyn}\dot{x}\left(t\right)=-x\left(t\right)+\tau_{m}\left(J_{syn}\sum_{syn}\delta \left(t-t_{syn}-\tau_{l}\right)\right)$		
**F–Input**			
**Type**	**Description**		
Poisson	“Thalamic”: time-constant input with rate λ.		
Poisson	“Long range cortico-cortical”: Ornstein Uhnlenbeck process (*OU*) with zero mean		
	$\tau_{n}\dot{OU}=-OU+\sigma \left(2\tau_{n}\right)W$		
	where W is a white noise process with zero mean		
**G–Measurements (for each population)**			
**Type**	**Description**		
Firing rate	Sum of spikes		
AMPA	Sum of AMPA PSCs (cortical and thalamic)		
GABA	Sum of GABA PSCs		
V_m_	Mean of membrane potential		
ΣI	Sum of AMPA and GABA PSCs. Note that AMPA and GABA have opposite signs.		
ΣI|	Sum of absolute values of AMPA and GABA PSCs.		
Weighted Sum (WS)	$\left[\sum_{pyr}AMPA\left(t-\tau_{AMPA}\right)-\alpha \sum_{pyr}GABA\left(t-\tau_{GABA}\right)\right]$		
Reference Weighted Sum (RWS)	$\left[\sum_{pyr}AMPA\left(t-6\;ms\right)-1.65\left(\sum_{pyr}GABA\left(t\right)\right)\right]$		

**Table 2:** Parameters for the two cell types used in the LIF network model.

Leaky integrate and fire model parameters	Pyramidal neurons	Interneurons
**Population**		
Size	4000	1000
**Connectivity**		
p_dc_	0.2	0.2
**Neuron**		
V_thr_ (mV)	18	18
V_reset_(mV)	11	11
*τ*_*m*_ (ms)	20	10
*τ*_*refractory*_ (ms)	2	1
**Synapse**		
*τ*_*rGABA*_ (ms)	0.25	0.25
*τ*_*dGABA*_ (ms)	5	5
*τ*_*rAMPA*_ (ms)	0.4	0.2
*τ*_*dAMPA*_ (ms)	2	2
*J*_*GABA*_ (mV)	-1.7	-2.7
*J*_*AMPA*−*cort*_. (mV)	0.42	0.7
*J*_*AMPA*−*th*_ (mV)	0.55	0.95
**Inputs**		
Thalamic input (spikes/ms)	[0.5:0.5:3, 6]	[0.5:0.5:3, 6]
OU τ_n_ (ms)	16	16
OU σ(mV)	0.25	0.25

The LFP signal that would result from the time series of spikes generated by the LIF network provided the postsynaptic neurons had biologically plausible dendritic structures, was computed by injecting the LIF synaptic activity into a 3D network of morphologically detailed multi-compartmental model neurons (Fig 1B, see Methods). A summary of the properties of the 3D network is reported in Table 3, while the synaptic parameters are listed in Table 4 (see Methods for further details). In order to set up the 3D network we were required to make additional assumptions regarding the spatial positioning of cells, the shape and size of their dendritic structures, as well as the synaptic distributions. We focused on computing the LFP generated by one cortical layer (in terms of soma positions) that comprised both inhibitory and excitatory neurons. In our default setting, we assumed all neurons in the 3D network to be inside two cylinders with 250 μm radius and 250 μm height that were stacked one above the other to resemble the vertical structure of layer 2/3 (Fig 1B). Note that this spatial scale is similar to the size of the neuronal pool contributing to the recorded LFP, the so-called spatial reach, in the case of uncorrelated synaptic activity driving a neuronal population [20,21,35], and resulted in a neuronal density consistent with known estimates of 50000 neurons per mm^3^ in the cortex [48]. While our two model populations most directly resemble a pair of excitatory and inhibitory populations in cortical L2/3, we show in subsection “Dependency of the LFP signal on dendritic morphology” that our results also pertain to the LFP generated by neuron morphologies found in other cortical layers.

**Table 3:** Summary of 3D network model of multi-compartmental neurons.

**A– 3D morphological network model summary**	
**Populations**	Populations of pyramidal cells and interneurons
**Neuron**	Passive multi-compartment neuron models
**Connectivity**	-
**Synapse model**	Bi-exponential functions defined by rise and decay time.Current-based synapses in all analysis except Fig 10 (conductance-based synapses)
**Input**	Synaptic input identical to the LIF neurons in the network model (Table 1)
**Measurements**	Model LFP signal, dipole moment
**B–Populations**	
Type	Populations of *N*_*e*_ pyramidal neurons and *N*_*i*_ interneurons
Geometry	Two cylinders with radius *R* separated in depth by distance *d*
Cell positions	Random soma positions within the lower cylinder, dendrites extending both cylinders
Parameters	*N*_*e*_, *N*_*i*_, *R*, *d*
**C–Connectivity**	
No network connectivity, but synaptic inputs derived from LIF network connectivity (Table 1)	
**D–Neuron**	
Type	Multi-compartmental models with unique dendritic morphologies
Morphology	Generated uniquely for each cell from distribution of synaptic contacts with axons from presynaptic cells (see Methods). For pyramidal cells synaptic contacts are distributed in both cylinders while interneurons make synaptic contacts only in lower cylinder.
Neuron dynamics	Non-spiking neurons with passive membrane with specific membrane resistance *R*_*m*, *pyr/int*_, specific axial resistance *R*_*a*_, and specific membrane capacitance *C*_*m*_
Compartments	Length of each compartment during simulation set to be shorter than the electrotonic length at 100Hz.
Parameters	*R*_*m*, *pyr/int*_, *R*_*a*_, *C*_*m*_
**E–Synapse**	
**Type**	**Bi-exponential current synapse (everywhere but Fig 10)**
**Description**	An incoming spike at t_syn elicits a postsynaptic current (PSC) for times t>t_syn:
	$PSC\left(t\right)=J_{syn}A\left[exp\left(\frac{-\left(t-t_{syn}\right)}{\tau_{decay}}\right)-exp\left(\frac{-\left(t-t_{syn}\right)}{\tau_{rise}}\right)\right]$ where A is a normalization factor to give a peak current J_syn_
**Type**	**Bi-exponential conductance synapse (Fig 10)**
**Description**	An incoming spike at t_syn_ elicits a postsynaptic current (PSC) for times t>t_syn:
	$PSC\left(t\right)=G\left(V\left(t\right)-E_{syn}\right)A\left[exp\left(\frac{-\left(t-t_{syn}\right)}{\tau_{decay}}\right)-exp\left(\frac{-\left(t-t_{syn}\right)}{\tau_{rise}}\right)\right]$ where A is a normalization factor to give a peak conductance G
Parameters	*τ_rise,_* *τ*_*decay*,_ *J*_*syn*,_ *G*, *E*_*syn*_ for each connection type
**F–Input**	
**Type**	**Description**
Thalamic	Time-constant rate *r*_*exc*_ as defined in the LIF network
Long-range cortico-cortical	Ornstein-Uhlenbeck process with rate *r*_*cc*_
Recurrent excitatory inputs	Recreated using the connectivity combined with the output spike trains in the LIF network and the output spikes from the excitatory LIF network population.
Recurrent inhibitory inputs	Recreated using the connectivity combined with the output spike trains in the LIF network and the output spikes from the inhibitory LIF network population.
Parameters	*r*_*exc*_, *r*_*cc*_
**G–Measurements**	
**Model LFP**	
Type	Extracellular field potentials calculated using the line-source method [31] as implemented in the LFPy toolbox [34].
Assumptions	Extracellular medium assumed to be purely resistive (non-capacitive, infinite volume) with extracellular conductivity *σ*_*cond*_.
Electrode placement	Ideal point-electrode (no filtering) placed in the center of the population at different electrode depths *z*_*elec*_
Parameters	*σ*_*cond*_, *z*_*elec*_
**Dipole moment**	
Type	Current dipole moment *d*_*z*_ along the *z*-direction computed for each point in time by a weighted sum of transmembrane currents: *d*_*z*_(*t*) = ∑_*i*_ *z*_*i*_ *I*_*i*_(*t*)
	where *I*_*i*_ is the transmembrane current in compartment *i* located at position *z*.

**Table 4:** Parameter values for the 3D network model of multi-compartmental neurons.

**A—Population**		
**Name**	**Description**	**Value**
*N*_*e*_	Number of pyramidal neurons	4000
*N*_*i*_	Number of interneurons	1000
*R*	Population radius	250 μm
*D*	Distance between cylinders used when generating morphologies	0–500 μm
**B–Neuron**		
**Name**	**Description**	**Value**
*R*_*m*, *pyr*_	Specific membrane resistance, pyramidal cells	30 kΩcm^2^
*R*_*m*, *int*_	Specific membrane resistance, interneurons	20 kΩcm^2^
*R*_*a*_	Specific axial resistance	150 Ωcm
*C*_*m*_	Specific membrane capacitance	1.0 μF/cm^2^
**C–Synapse**		
**Excitatory, pyramidal cells ->pyramidal cells**		
**Name**	**Description**	**Value**
*τ*_*rise*_	Synaptic rise time constant	0.4 ms
*τ*_*decay*_	Synaptic decay time constant	2 ms
*J*_*syn*_	Synaptic weight (current based)	0.070 nA
*G*	Synaptic weight (conductance based)	0.014 μS
*E*_*syn*_	Synaptic reversal potential (conductance based)	0 mV
**Excitatory, thalamic ->pyramidal cells**		
**Name**	**Description**	**Value**
*τ*_*rise*_	Synaptic rise time constant	0.4 ms
*τ*_*decay*_	Synaptic decay time constant	2 ms
*J*_*syn*_	Synaptic weight (current based)	0.091 nA
*G*	Synaptic weight (conductance based)	0.0027 μS
*E*_*syn*_	Synaptic reversal potential (conductance based)	0 mV
**Excitatory, external cortical -> pyramidal cells**		
**Name**	**Description**	**Value**
*τ*_*rise*_	Synaptic rise time constant	0.4 ms
*τ*_*decay*_	Synaptic decay time constant	2 ms
*J*_*syn*_	Synaptic weight (current based)	0.070 nA
*G*	Synaptic weight (conductance based)	0.014 μS
*E*_*syn*_	Synaptic reversal potential (conductance based)	0 mV
**Inhibitory, interneurons ->pyramidal cells**		
**Name**	**Description**	**Value**
*τ*_*rise*_	Synaptic rise time constant	0.25 ms
*τ*_*decay*_	Synaptic decay time constant	5 ms
*J*_*syn*_	Synaptic weight (current based)	-0.145 nA
*G*	Synaptic weight (conductance based)	0.0057μS
*E*_*syn*_	Synaptic reversal potential (conductance based)	-90 mV
**Excitatory, pyramidal cells ->interneurons**		
**Name**	**Description**	**Value**
*τ*_*rise*_	Synaptic rise time constant	0.2 ms
*τ*_*decay*_	Synaptic decay time constant	1 ms
*J*_*syn*_	Synaptic weight (current based)	0.093 nA
*G*	Synaptic weight (conductance based)	0.0023 μS
*E*_*syn*_	Synaptic reversal potential (conductance based)	0 mV
**Excitatory, thalamic ->interneurons**		
**Name**	**Description**	**Value**
*τ*_*rise*_	Synaptic rise time constant	0.2 ms
*τ*_*decay*_	Synaptic decay time constant	1 ms
*J*_*syn*_	Synaptic weight (current based)	0.126 nA
*G*	Synaptic weight (conductance based)	0.0047 μS
E_syn_	Synaptic reversal potential (conductance based)	0 mV
**Excitatory, external cortical ->interneurons**		
**Name**	**Description**	**Value**
*τ*_*rise*_	Synaptic rise time constant	0.2 ms
*τ*_*decay*_	Synaptic decay time constant	1 ms
*J*_*syn*_	Synaptic weight (current based)	0.093 nA
*G*	Synaptic weight (conductance based)	0.023 μS
*E*_*syn*_	Synaptic reversal potential (conductance based)	0 mV
**Inhibitory, interneurons ->interneurons**		
**Name**	**Description**	**Value**
*τ*_*rise*_	Synaptic rise time constant	0.25 ms
*τ*_*decay*_	Synaptic decay time constant	5 ms
*J*_*syn*_	Synaptic weight (current based)	-0.092 nA
*G*	Synaptic weight (conductance based)	0.090 μS
*E*_*syn*_	Synaptic reversal potential (conductance based)	-90 mV
**D–Measurements**		
**Model LFP**		
**Name**	**Description**	**Value**
*σ*_*cond*_	Extracellular conductivity	0.3 S/m
*z*_*elec*_	Electrode depth	(-400)– 400 μm, in steps of 25 μm

Given these geometrical constraints, we created the multi-compartmental cell models in the 3D network in the following way: soma locations for all cells were homogeneously distributed within the lower cylinder (Fig 1B). Next, we placed artificial straight axons that were distributed at random cortical depths and random orientations within both cylinders. They served as targets in an algorithmic generation of dendrites, through which pyramidal cell dendrites were connected to all axons within a specified reach distance while optimizing the following wiring conditions: short conductions times, short total cable length and synaptic democracy (i.e., equal impact of synaptic inputs at the site of dendritic integration [49,50]). This procedure has been shown previously to reproduce pyramidal-cell-like dendrites [51]. The number of axons and their length were set so that the resulting cell morphologies matched the membrane surface distribution of real cortical layer 2/3 pyramidal cell reconstructions [52] within the constraints of the simplified columnar arrangement that was chosen for this study. This procedure also provided good matches for total cable lengths and number of branch points (compare membrane surface distribution in S1 Fig and see Methods for more details). Note that the virtual axons used for the generation of the morphologies were subsequently discarded. Since the membrane area (and consequently the transmembrane current) of the axons is very small compared to the dendrites, we expected them to have a negligible contribution to the present 3D network LFP generation.

Stellate cell dendrites were generated in a similar manner, but were only connected to axons in the lower cylinder. This resulted in stellate cell morphologies with realistic bush-like dendrites. Fig 1C illustrates the overall structure of the resulting 3D network.

To further validate the simulation results obtained with these morphologies, we also built an alternative 3D network with anatomically reconstructed morphologies (see Methods) and checked that the results were essentially the same as for algorithmically grown morphologies (see subsection “Performance of LFP proxies in different dynamic network states”).

Finally, AMPA synapses were homogeneously distributed over the whole neuronal surface while GABA synapses were located only in the lower cylinder, closer to the soma (Fig 1B, see Methods for details). Alternative synaptic distributions are explored in the “Dependency of the LFP signal on the distribution of synapses” subsection.

For each neuron in the LIF network we randomly assigned a multi-compartmental neuron model with a unique dendritic structure in the 3D network. The connectivity of the LIF network determined which postsynaptic spikes in the LIF network simulation should serve as input spikes for each multi-compartment neuron. We then used these spike times together with the external input (see above) to activate synaptic currents in the 3D network (see Methods). In this way we assured that the synaptic input in a multi-compartment neuron was identical to its LIF neuron counterpart. The synaptic dynamics in the 3D network was identical to that in the LIF network. In a subsequent step, we took into account all transmembrane currents in the neurons of the 3D network to compute the LFP by means of well-established volume conduction theory and the so called line-source method [31,34] (see Methods).

### Spatial distribution of simulated LFP signal

Fig 1 shows a half-second excerpt of results for an example simulation using the spiking activity generated by the LIF network (Fig 1D) in response to a 1.5 spikes/ms stimulus (see Methods for details) to calculate the corresponding LFP signal along the vertical axis of the cylinder at different electrode depths from the 3D network (Fig 1E). The temporal fluctuations of the LIF signal were strongly correlated across depth, albeit with a sign shift around the depth just between the two cylinders (which we from now on will refer to as the inversion point). The sign of the baseline (DC) LFP was negative above the inversion point while it was positive below it. This reflects that the LFP was dominated by the perisomatic inhibitory synapses generating a net source current close to the soma and sink return currents in the apical branches. The excitatory synapses contributed less due to their homogeneous distribution (Fig 1B), giving only a weak current dipole [29], as will be discussed in more detail in the next sections.

**Fig 2:**
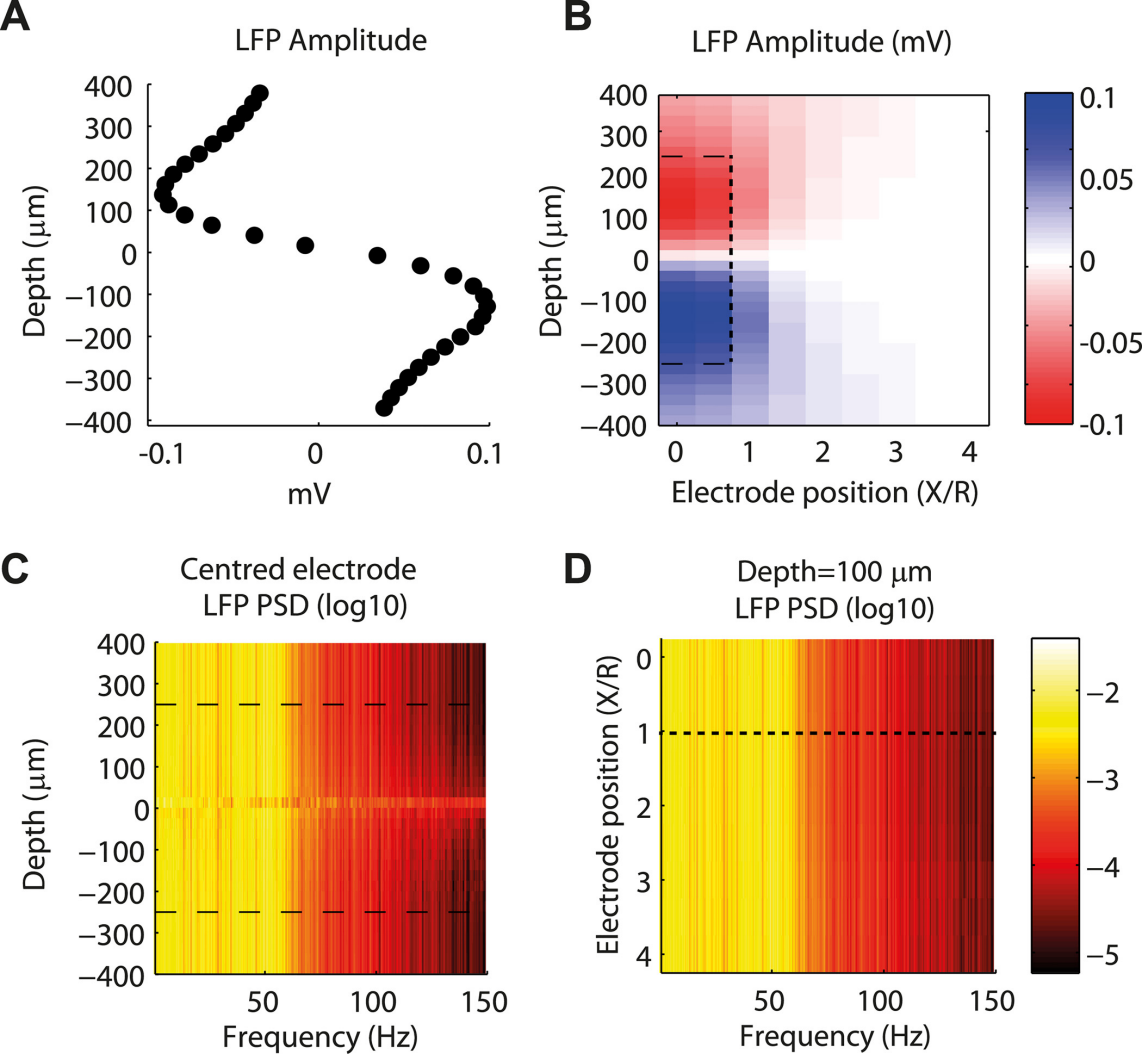
Simulated local field potentials (LFPs) as a function of depth and lateral position of the electrode from the center of the 3D network. (A) Amplitude of LFP signal generated by the morphological 3D network at 50 μm spaced depths, when the dynamics were driven by a 10 seconds thalamic input of 1.5 spikes/ms. The amplitude was measured as the standard deviation of the LFP signal over the entire time course with the same sign as the baseline (see Fig 1E). Dashed lines indicate network boundaries (-250 μm < d < 250 μm) (B) Amplitude of LFP signal at different depths and distances from the center of the 3D network. Distances were measured in units of 3D network radius *R* (= 250 μm). The dashed lines separate the area inside the network (*X*/*R*≤1 & -250 μm < d < 250 μm) and outside the network. (C) Power spectral density (PSD) of LFP signal in the center of the 3D network for different depths. Dashed lines indicate network boundaries -250 μm < d < 250 μm). (D) Power spectral density (PSD) of LFP signal at different distances from the center of the 3D network at a reference depth of 100 μm. Dashed line indicates network boundary (*X*/*R* = 1).

We defined the amplitude of LFP fluctuations at each depth as the standard deviation of the signal over time, and further assigned it the same sign as the LFP baseline, i.e., negative/positive above/below the inversion point. The magnitude of the LFP amplitude was largest around the middle of each cylinder (Fig 2A), decreased steeply close to the inversion point and more smoothly beyond the vertical boundaries of the network. The decrease of the amplitude of the LFP fluctuations when the electrode was moved away from the center of the 3D network is shown in Fig 2B for all depths. This decrease in LFP power was consistent with results of [20]: inside the 3D network (*X*/*R* < 1, where *X* is the displacement of the electrode from the center and *R* is the radius of the cylinder) differences were small, but when the electrode was placed outside the 3D network (*X*/*R* > 1) the decrease was steep. Note that the region around the inversion point where the potential is very small, broadened with the distance from the center.

We observed that all power spectra recorded outside this noise-dominated region had similar shapes (Fig 2C and 2D), suggesting that LFP fluctuations could be roughly approximated by the same time series rescaled by the numerical value of the LFP amplitude shown in Fig 2B. The observation that LFPs recorded in different spatial positions had similar temporal behavior and differed mainly by a scaling factor, suggested that a single LFP proxy could work for recordings at different depths and positions in the horizontal plane, provided that it is properly scaled. Such a factorization of spatial and temporal dimensions can be expressed (see [30]) as

$$LFP_{proxy}\left(r,d,t\right)=f_{proxy}\left(r,d\right)*g_{proxy}\left(t\right)$$ (1)

where *d* is the depth and *r* the distance from the population center. The term *f*_*proxy*_(*r*, *d*) then gives the amplitude of the signal as a function of the electrode position (as in Fig 2B) while the dimensionless *g*_*proxy*_(*t*) has variance equal to one and describes the temporal features of the LFP signal.

We first focused on finding the optimal *g*_*proxy*_(*t*) for an LFP signal recorded at selected depths along the central vertical axis (*X/R = 0*) of the 3D network. However, we found (see subsection “New class of LFP proxies”) that the identified optimal LFP proxy was applicable also to other depths and radial distances of the populations (given an appropriate overall scaling of the signal amplitudes, cf. Fig 2B).

**Fig 3:**
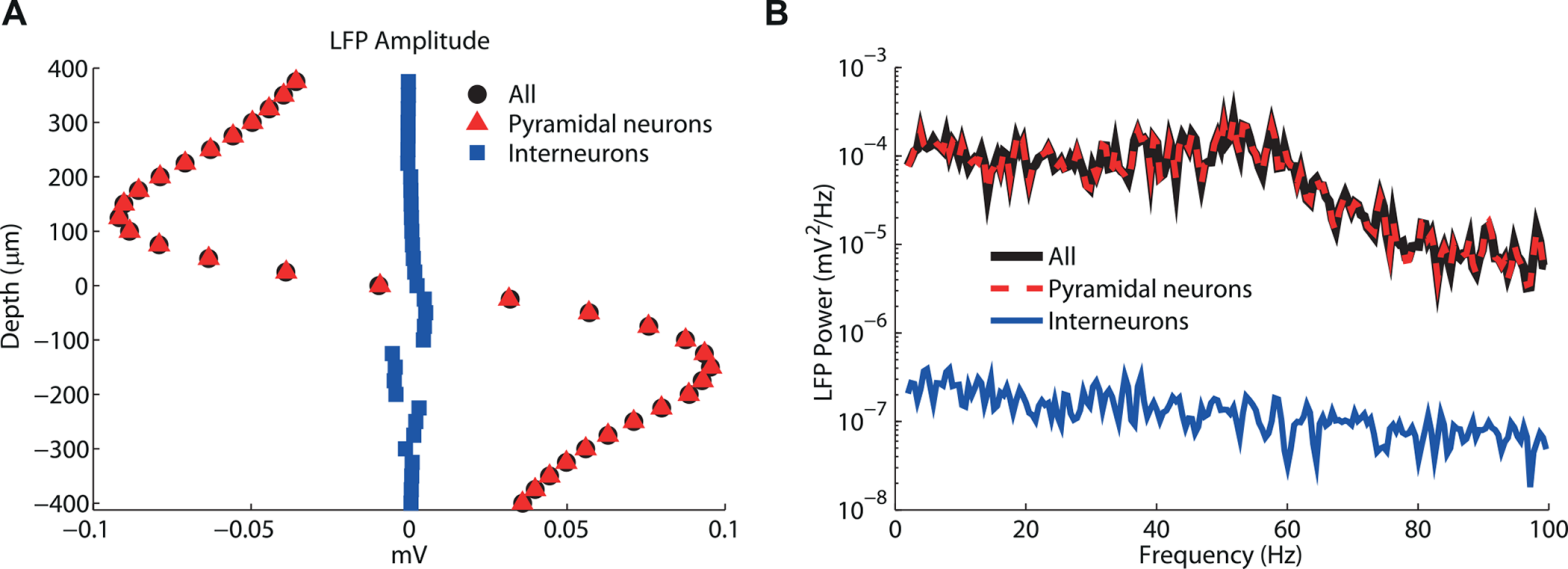
Contribution from individual neuron types to simulated LFP signal. Decomposition of LFP obtained in same conditions as Fig 2 into contributions from currents through the membrane of interneurons and pyramidal neurons. (A) Depth-resolved amplitude of LFP signal generated by all neurons (black), by the pyramidal neurons (red), and by the interneurons (blue). (B) Corresponding LFP power spectra for the three sets depicted in (A) at a depth of 100 μm.

The contribution to the LFP signal from synaptic inputs onto the interneurons (and their associated return currents) was negligible both in amplitude (Fig 3A) and in determining the LFP spectrum (Fig 3B). This was due to the different morphologies of the two types of neurons: consistently with what was shown previously for stellate cells with symmetrically placed synapses [20] (i.e., a so-called close-field arrangement [53]), the contribution from the interneurons to the LFP was negligible (Fig 3). Further, the associated power spectrum of this contribution was closer to colored noise and did not display gamma fluctuations. We investigate this in detail in the subsection “Dependency of the LFP signal on dendritic morphology”.

Since we obtained a very similar LFP when we only simulated the contribution from synaptic inputs onto the pyramidal neurons, all the results shown in the following will, unless otherwise stated, consider only the contributions from pyramidal neurons to LFP. Likewise, the LFP proxies will be based only on input onto excitatory neurons (as done previously [15]). However, the inhibitory neurons obviously play a key role (i) in generating the dynamics and (ii) in providing the GABA currents of synapses onto pyramidal neurons that contribute strongly to the LFP.

### Performance of different LFP proxies

**Fig 4:**
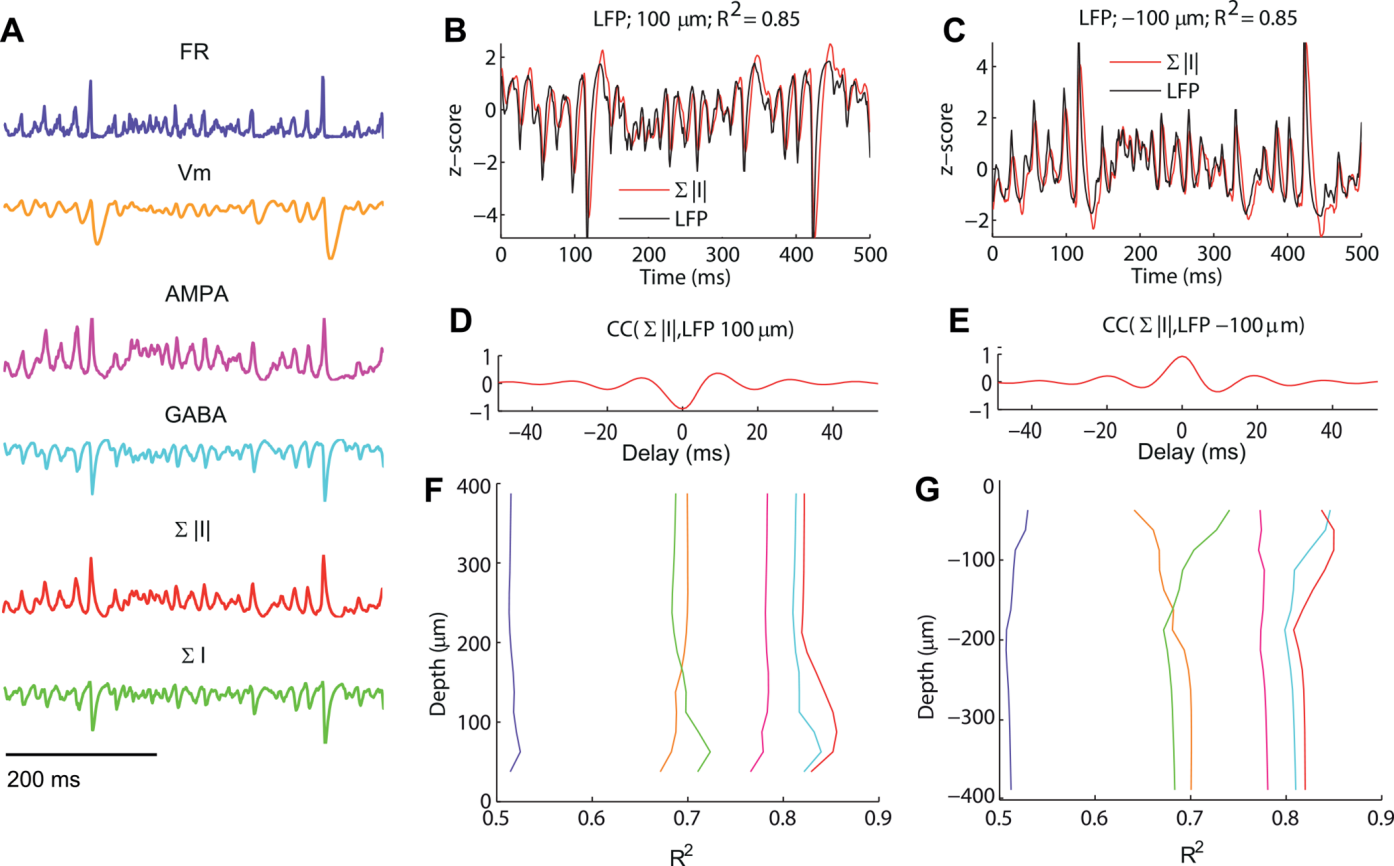
Performance of candidate LFP proxies. (A) Illustrations of predictions of LFP time courses from candidate LFP proxies. From top to bottom: firing rate (FR), membrane potential (V_m_), AMPA currents, GABA currents (note: these have a negative sign), sum of absolute values of AMPA and GABA currents ∑|*I*|, sum of AMPA and GABA currents ∑*I*. Results are shown for a thalamic stimulation of 1.5 spikes/ms, and the proxies are normalized to have variance equal to one (see text),. (B-C) time course of the LFP signal (black) for reference depths 100 μm (B) and -100 μm (C) compared to the best matching proxy, ∑|*I*| (magenta). The title indicates the fraction of variance explained (85% in both cases). (D-E) Cross-correlation in time between the LFP and the ∑|*I*| proxy for the two depths. Note that the peaks corresponding to the highest cross-correlation magnitudes corresponded to a lag of 1 ms, i.e., the LFP was best predicted by the value of ∑|*I*| one millisecond in the past. (F-G) Fraction of LFP signal variance explained by different LFP proxies with optimal delay (same color code as (A)). as a function of depth. The sum of absolute values of the synaptic currents ∑|*I*| was the best proxy, followed by the use of GABA alone. The firing rate FR was a poor proxy, and the other three were moderately good proxies.

We first tested six LFP proxy candidates (Fig 4A): AMPA currents, GABA currents, the average firing rate FR, the average membrane potential V_m_, and the sum of these synaptic currents ∑*I* as well as their absolute values ∑|*I*|. Note that the "AMPA currents" and "GABA currents" proxies are defined as the sum of the post-synaptic currents for each type of synapse over all pyramidal neurons (see Table 1G). These currents have depolarizing and hyperpolarizing effects, respectively, on the postsynaptic neurons. We thus here use the convention that assigns a positive sign to AMPA currents and a negative sign to GABA currents.

Because of the opposite signs assigned to the AMPA and GABA currents, the sum of the absolute values of the currents ∑|*I*| is equivalent to the difference between the currents. For several reasons, i.e., synaptic delay and dendritic filtering, we expected the best proxy for the LFP time course to possibly involve time-delayed measures of LIF network variables. To assess the best values of these delays we first computed the cross-correlation function between the ground-truth LFP and the considered LFP proxy obtained from the LIF network, and found the delay at which the absolute value of the correlation was largest (for half of the recording depths the correlation is negative due to LFP inversion). The LFP proxy that we chose was the z-scored (i.e., baseline-subtracted and normalized to have variance equal to one) and time-shifted LIF network variable that maximized the fraction of variance explained, R^2^. Finding the best delay and rescaling factor was done separately for each depth, but we found that the differences in the observed best values of the delay across depth, were minor (see S2B Fig). Fig 4B and 4C shows the comparison between the 3D network LFP signal at two different electrode depths and the LFP proxy given by the sum of absolute values of the synaptic currents ∑|*I*|, that given our sign convention simply becomes the difference between the currents, i.e.,

$$LFP_{\sum \left|I\right|}\left(r,d,t\right)=f_{\sum \left|I\right|}\left(r,d\right)*Norm\left[\sum_{pyr}AMPA\left(t-\tau \right)-\sum_{pyr}GABA\left(t-\tau \right)\right]$$ (2)

where *Norm*[] indicates the mean-subtracted, normalized version of the time series between square brackets. Fig 4D and 4E shows the cross correlation between the 3D network LFP signal and proxy for the two depths.

A comparison of the average fraction of variance explained by all the LFP proxies displayed in Fig 4A across different depths (Fig 4F and 4G) shows that the best one was the sum of absolute values of synaptic currents ∑|*I*| (<R^2^> = 0.83) followed by the negative of the GABA currents (<R^2^> = 0.81) and then the AMPA currents (<R^2^> = 0.78). The negative of the sum of synaptic currents ∑*I* and membrane potential V_m_ performed in a similar way (<R^2^> = 0.69), while the firing rate FR gave a poor fit (<R^2^> = 0.51). The R^2^ is slightly larger for depths about 100 μm from the inversion point, probably due to stronger synaptic and return currents.

We found two results to be of particular interest. The first was that a proxy based on GABA currents alone gave clearly a better match for the simulated LFP signal than the AMPA currents alone. The second was that the ∑|*I*| gives the best fit which suggests that the magnitude of the AMPA currents locally sums with the magnitude of the GABA return currents. Thus the two types of synaptic currents contribute to the LFP with the same sign. This feature is partly due to the fact that AMPA synapses are distributed over the whole surface of pyramidal neurons, while GABA synapses are located only in the lower cylinder close to the soma (Fig 1B). This will be further investigated in the “Dependency of the LFP signal on the distribution of synapses” subsection.

The fits above were computed by averaging the time-varying variables over the set of excitatory neurons in the LIF network. However, we also tested the quality of the fit obtained by averaging over all the neurons in the LIF network or only over inhibitory neurons. The results for each variable and depth are shown in S2A Fig, together with the associated optimal delays. The relative ranking of the candidate proxies remains unaltered. Further, proxies obtained by averaging the firing rate, the membrane potential, or the synaptic input currents over the excitatory neurons (as above) performed better than proxies obtained by averaging the same variables over the inhibitory neurons set and roughly the same as proxies obtained averaging over all neurons (S2A Fig).

### New class of LFP proxies

Since AMPA and GABA currents contributed differently to the LFP signal we investigated a novel proxy, the *weighted sum between AMPA and GABA currents* (WS), that uses a linear combination of AMPA and GABA synaptic currents where we introduce a factor *α* describing the relative contribution of the two currents and a specific delay for each type of current:

$$LFP_{WS}\left(r,d,t\right)=f_{WS}\left(r,d\right)*Norm\left[\sum_{pyr}AMPA\left(t-\tau_{AMPA}\right)-\alpha \left(\sum_{pyr}GABA\left(t-\tau_{GABA}\right)\right)\right]$$ (3)

Note that the two proxies ∑|*I*| and ∑*I* are particular cases of the above equation in which the delays are the same, and *α* is equal to 1 and -1 respectively.

**Fig 5:**
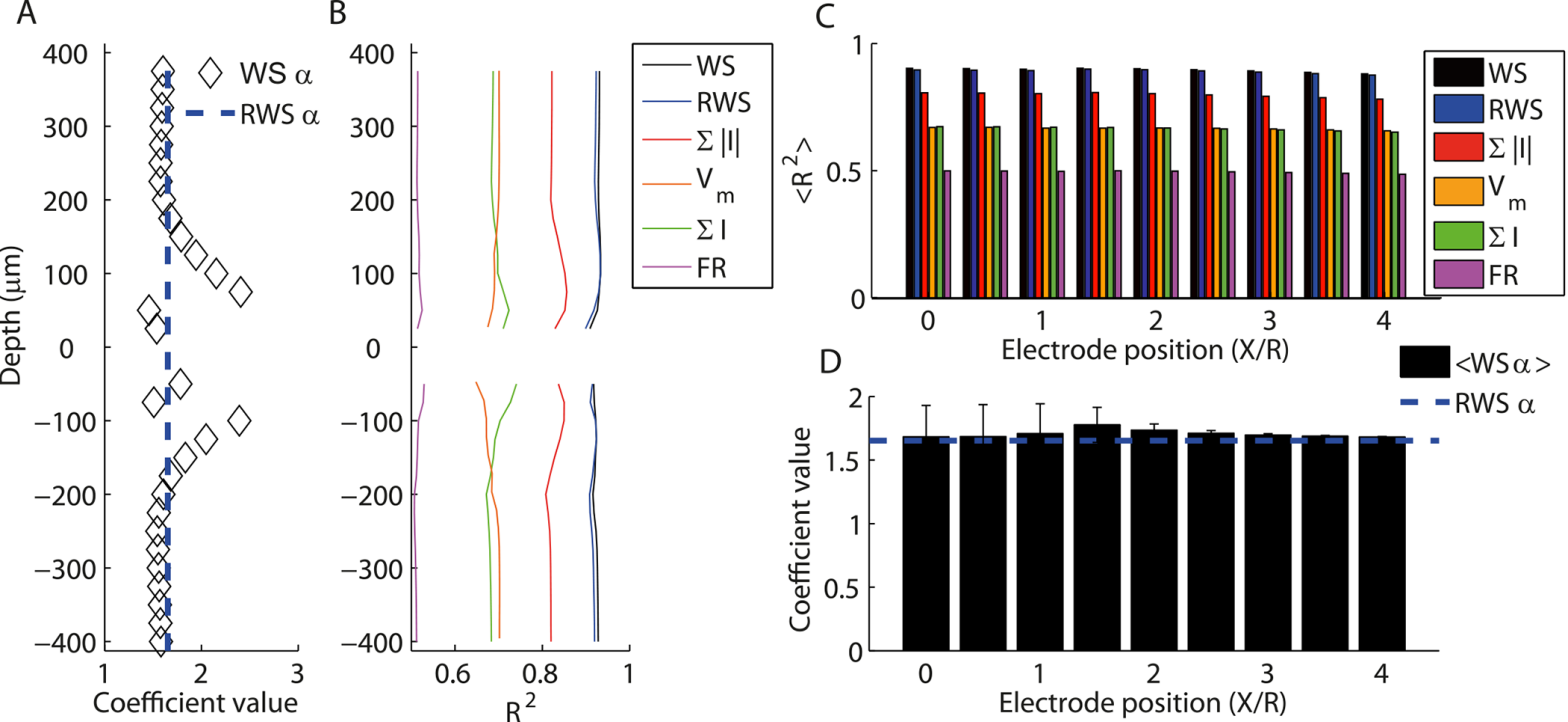
New proxy explaining more than 90% of the variance in the LFP signal. (A) Value of relative contribution of AMPA and GABA currents (α parameter in Eq (3)) optimizing correlation between WS proxy and ground truth LFP in the same conditions as Fig 4. Dashed line indicates average value over depths α = 1.65 used for RWS proxy (Eq 4). (B) Same as Fig 4F and 4G including also WS proxy (black) and RWS proxy (blue). (C) Fraction of LFP variance explained, averaged over all depths, by different proxies for different distances from the center of the 3D network. From best to worst: weighted sum (WS), reference weighted sum (RWS), sum of absolute values of the synaptic currents (∑|*I*|), membrane potential (V_m_), sum of synaptic currents (∑*I*), firing rate (FR). Error bars are not displayed since they would not be visible in the figure. (D) Mean and standard deviation across depths of optimal coefficients of α in the WS proxy as a function of electrode lateral position. Dashed line indicates the fixed coefficient of the RWS proxy that was not optimized but kept constant for all depths and distances.

We first tested the WS proxy with the electrode located in the center of the 3D network for different depths. The optimal value of *α* was always positive, but varied across depths (Fig 5A). The optimal delays were always in the range [5–7] ms for *τ*_*AMPA*_ ms and in the range [-1 1] ms for *τ*_*GABA*_. This implies that the optimal LFP proxy was achieved by subtracting the GABA PSCs (postsynaptic currents) from the AMPA PSCs occurring around 6 ms in the past. Performance was very high for all depths (up to 93% of variance explained, see Fig 5B). Since the optimal values of *α*, (Fig 5A) *τ*_*AMPA*_ and *τ*_*GABA*_ (S2B Fig) were relatively stable across depths, we defined a new proxy: the *reference weighted sum LFP proxy* (RWS). The structure of the RWS proxy is the same as the WS proxy but the variables are fixed: *α* is set to the average accross depths of the optimal values for WS (1.65, see Fig 5A) and the delays to *τ*_*AMPA*_ = 6 ms and *τ*_*GABA*_ = 0 (S2C Fig). This results in

$$LFP_{RWS}\left(r,d,t\right)=f_{RWS}\left(r,d\right)*Norm\left[\sum_{pyr}AMPA\left(t-6\;ms\right)-1.65\left(\sum_{pyr}GABA\left(t\right)\right)\right]$$ (4)

We found that the performance of this proxy was almost indistinguishable from the single-depth optimized values across depths (Fig 5B) and largely outperformed all other proxies. Moreover, we found the performance of a proxy with *α* = 1.65 to be very good (>80% of variance explained) for a broad range of other AMPA- and GABA-current delays (S2C Fig).

We next tested the performance of the proxies for different distances of the electrode from the center of the 3D network: Fig 5C compares the fraction of variance explained by WS, RWS and the other proxies mentioned above for LFPs measured at different distances from the center of the 3D network. The depicted results are found from averaging across all depths. The Standard Error of the Mean of R^2^ across depths was <1% for all proxies and all lateral displacements and is not displayed in the figure since it would not be visible. Values for explained variance were very stable for different lateral electrode positions: in particular, for all lateral displacements RWS performances were similar to WS and outperformed all other proxies (Fig 5C). The average optimal value of *α* across depths was always close to the reference value 1.65 (Fig 5D). Given that the RWS proxy was much simpler than WS (see below) and able to explain more than 90% of the variance of the LFP time course at a wide range of electrode recording positions, we tentatively propose this as the best proxy for the LFP signal computable directly from LIF network variables.

The proxies given by the combination of two synaptic parameters (WS and RWS) have four free parameters (scale as described by the function f in Eq 1 and following, AMPA and GABA delays, relative amplitude of AMPA and GABA contribution) while the other proxies have only two free parameters (scale, delay). We assessed by means of the Bayesian Information Criterion (BIC, [54], see Methods for details) whether the benefit in terms of improved performance of the models based on the linear combinations of synaptic currents was worth the increase in model complexity due to the higher number of parameters. We found that, according to this model selection criterion, RWS outperforms all previous proxies and WS outperforms RWS and all other proxies (S3 Fig), demonstrating the power of the RWS and WS models. However, the optimal WS parameters are by construction different for each recording position and, as we will see in the following, for various network structures and states. Thus comparison of LFP predictions from use of the WS proxy requires detailed knowledge about recording position as well as the characteristics of the underlying network, and will thus have limited practical use. On the other hand, as the parameters of the RWS proxy are fixed, it can be used directly for all locations in space. As seen in the following, the RWS proxy performs well for a broad set of conditions (input intensity, neuron morphology, synaptic distribution), and this means crucially that the proxy can be used also under weak assumptions about the spatial structure of the underlying network. We thus conclude that RWS is the best LFP proxy based on LIF network variables. In the following we will test its robustness for different dynamic network states, spatial architectures and synaptic properties.

### Performance of LFP proxies in different dynamic network states

So far we investigated the LFP proxies using LIF networks in a state exhibiting weakly synchronized oscillations in the spiking dynamics, stimulating the LIF network at a relatively low intensity (1.5 spikes/ms). However, LIF networks can generate a variety of different dynamic network states when the frequency of external inputs is varied [14,15,18]. In order to test LFP proxies in different dynamic network states, we stimulated the LIF network with a wide range of input intensities, covering both much higher and much lower intensities than the one tested in above.

**Fig 6:**
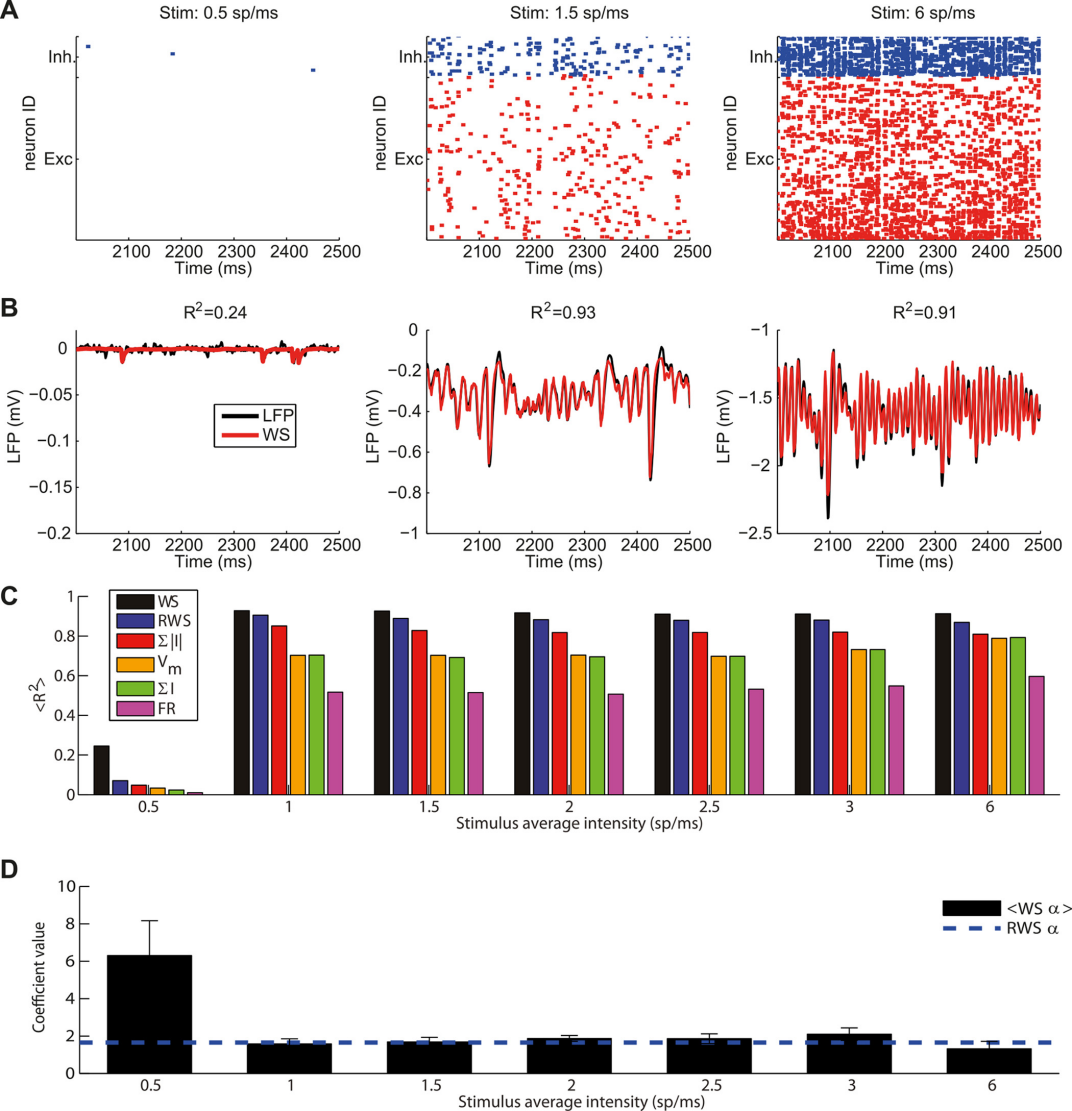
Effects of dynamic network states of the LIF model on the simulated LFP signal. (A) Raster plots of 50 interneurons (blue, top) and 200 pyramidal neurons (red, bottom). Neurons displayed are those with the highest number of spikes fired in the considered interval. Each panel corresponds to a different stimulation frequency: from left to right: 0.5 spikes/ms, 1.5 spikes/ms (the stimulation used in Figs 1–4), 6 spikes/ms. Note that in the selected interval all pyramidal neurons and most interneurons were silent for 0.5 spikes/ms stimulation. (B) LFP signal (black line) for 100 μm depth and corresponding best fit with the WS proxy (red) for these three stimulation frequencies. The titles show the fraction of variance explained over the whole 10 second simulation period. Note the different vertical scales. (C) Average fraction of LFP variance explained over all depths by different proxies for different thalamic input frequencies. Error bars are not displayed since they would not be visible in the figure. Same proxy arrangement as Fig 5C. (D) Mean and standard deviation across depths of optimal coefficients of α in the WS proxy as a function of thalamic input. Dashed line indicates the fixed coefficient of the RWS proxy.

Fig 6A shows, from left to right, a raster plot of a subset of neurons in the LIF network for a low-intensity input (0.5 spikes/ms), the default input level (1.5 spikes/ms), and a high-intensity input (6 spikes/ms). Shown below (Fig 6B) is the LFP signal generated in the 3D network at the reference depth of 100 μm for these three cases together with their corresponding WS fits. For external stimulation with 0.5 spikes/ms, recurrent activity in the LIF network was almost absent, with all pyramidal neurons and most interneurons being silent. The LFP amplitude was very small and the signal very noisy. For an input of 1.5 spikes/ms, firing was sparse with coexisting slow and high-frequency LFP fluctuations, and for 6 spikes/ms the dynamics were dominated by high-frequency LFP gamma oscillations also visible in the LIF network spiking activity.

With an input frequency of 0.5 spikes/ms, none of the candidate proxies was able to account for the LFP (Fig 6C). This was presumably because in these low-firing conditions, randomly occurring, uncorrelated synaptic inputs onto the neurons close to the electrode dominated the LFP signal. Such activity does not give a strong dipolar LFP pattern [21] and is apparently more difficult to capture with the global LIF network variables considered in the proxies. For the larger inputs ranging from 1 to 6 spikes/ms, however, the WS proxy was able to explain more than 91% of the variance. RWS was able to explain 88–91% of the variance between 1 to 3 spikes/ms with a small decrease to 87% for an input of 6 spikes/ms (Fig 6C). For inputs of 1 spikes/ms or more the sum of the absolute values of the synaptic currents explained 81–85% of the variance, the membrane potential 70–79%, the sum of the currents 70–79%, and the firing rate only 51–60%. Overall, the ranking of the proxies regarding their R^2^ values remained the same for all dynamic network states and the RWS provided an excellent proxy in all cases. As shown in Fig 6D the relative weighting between AMPA and GABA currents as given by the parameter *α* for the WS proxy was stable and close to the reference values 1.65 chosen for RWS for input stimulus intensities, except for the case of very low-intensity input in which the LFP signal is almost absent and the fit is poor.

**Fig 7:**
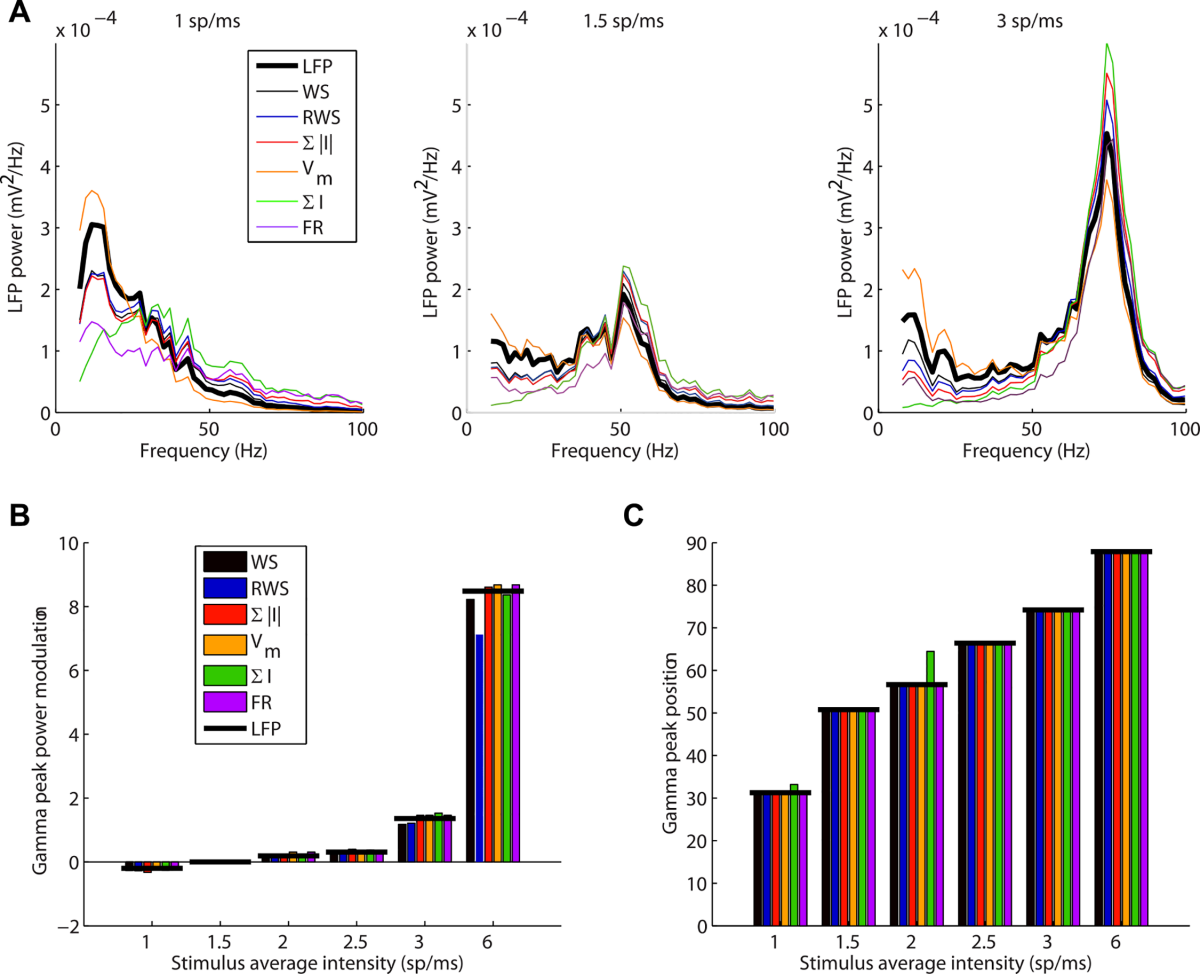
Spectral analysis of LFP signal. (A) Power spectra of the LFP signal at recorded at the 100 μm depth position, and predictions from candidate LFP proxies for input stimulation frequencies of 1 spikes/ms (left), 1.5 spikes/ms (center), 3 spikes/ms (right). Similar results are found for recordings at all depths. Note that for low inputs the power decreased almost monotonously with frequency, while for sufficiently strong input, LFP gamma fluctuations appeared and increased in power and frequency with stimulus intensity. (B) Variation of gamma (30–100 Hz) peak power with stimulus input intensity for the LFP signal and all proxies (measured as relative increase compared to the power at 1.5 spikes/ms). (C) Gamma peak frequency as a function of input frequency for the LFP signal and all proxies.

A key property of the LIF network is that it exhibits a prominent gamma-band activity (30–100 Hz) in the overall firing activity when the input intensity is increased as indicated by an increased peak in the power spectral density (PSD) [15]. We therefore investigated how this is reflected in our simulated LFP signal and how well the LFP proxies capture these properties of the LFP signal. Fig 7A shows the power spectra for three different input frequencies. All proxies except for the membrane potential tended to underestimate the low frequency LFP fluctuations and to overestimate frequencies in the gamma range. WS and RWS proxies both produced a nearly perfect fit of the LFP spectrum in the gamma-band range while exhibiting the smallest error in the low frequency components among all proxies. In the 1–3 spikes/ms input range the modulation of the LFP gamma power was well approximated by all proxies, while for 6 spikes/ms input, WS and RWS underestimated it (Fig 7B). All proxies essentially predicted the correct peak LFP gamma frequency (Fig 7C) for all input levels above 1 spikes/ms.

### Dependency of the LFP signal on dendritic morphology

**Fig 8:**
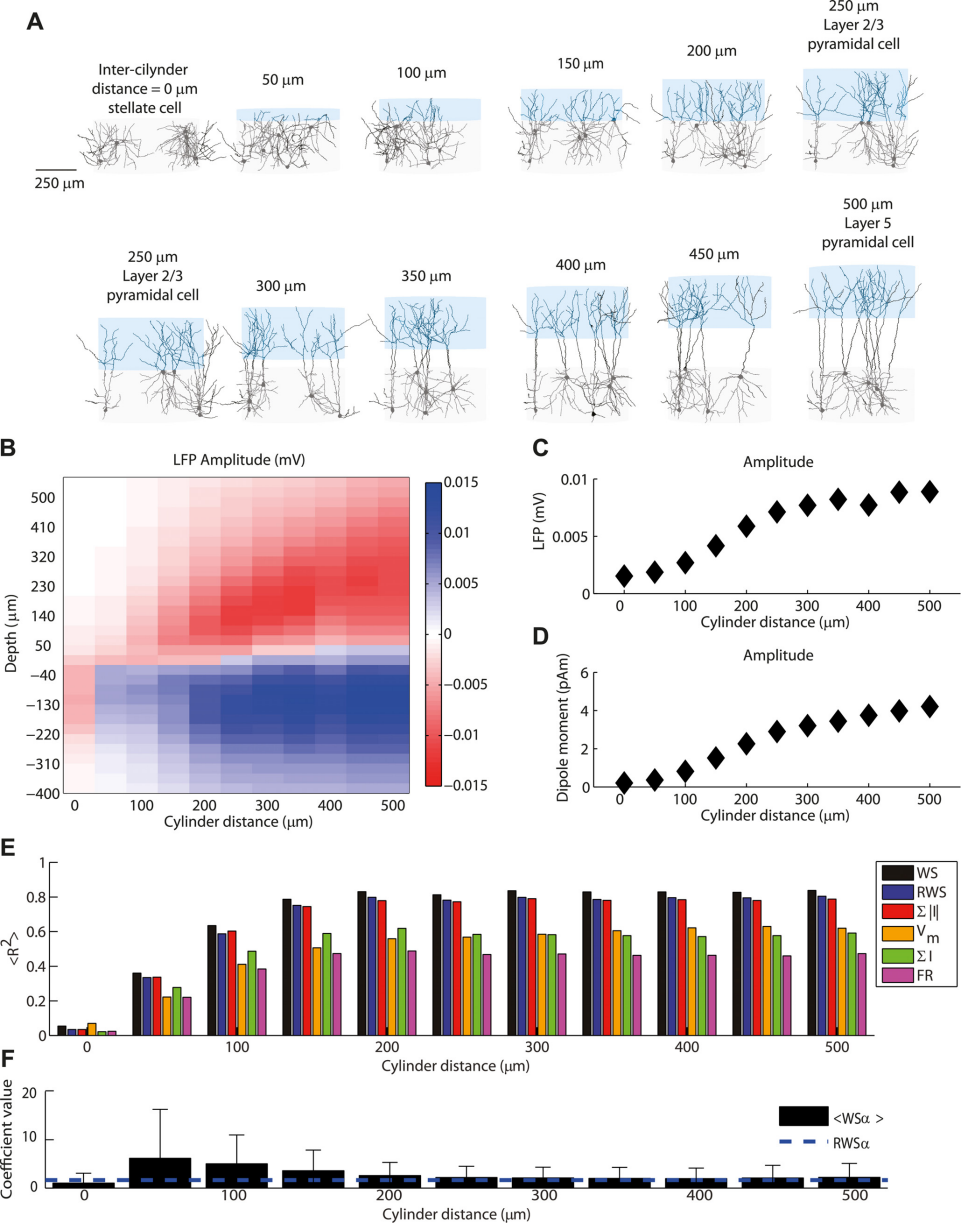
Effect of neuronal morphologies on neural signal. (A) Manipulation of the relative position of the two cylinders in which the dendrite arborizes, 50 μm steps. Each relative position induces a specific ‘pyramidalness’ of the model cortical cells (see Methods for details). Red and blue lines indicate the center of the upper and lower cylinder respectively. When the two cylinders are completely superimposed (first panel of top row), the structure corresponds to a stellate cell. When the two cylinders are on top of each other (last panel of top row, first of bottom row), the morphology corresponds to a layer 2/3 pyramidal cell. When the boundaries of the two cylinders are separated by 250 μm (last panel of bottom row), the cell morphology resembles a layer 5 pyramidal cell. (B) LFP amplitude as a function of depth and distance between the two cylinders. (C) Average absolute amplitude (standard deviation) over depths of LFP fluctuations as a function of distance between cylinders. (D) Amplitude of current dipole moment as a function of distance between cylinders. (E) Average fraction of LFP variance over all depths explained by different proxies for different distances between cylinders. Error bars are not displayed since they would not be visible in the figure. Same proxy arrangement as in Fig 5C. (F) Mean and standard deviation across depths of optimal coefficients of α in the WS proxy as a function of distance between cylinders. Dashed line indicates the fixed coefficient of the RWS proxy. Note that since for distances below 100 μm the performance of the fit was poor (see panel (E)), the fitted value of the relative weight of AMPA and GABA currents in contributing to the LFP signal has little significance.

We hypothesized that the negligible contribution of inhibitory neurons was due to the weak dipole moment created by the symmetrically placed synapses on the dendrites of stellate cells [29]. To test this hypothesis we investigated in the following the effect of neuron shape on the LFP generation by systematically altering the morphology of the interneuron population while keeping its inputs fixed. This manipulation also tested the robustness of the LFP proxies to the specific choice of the neuronal morphology. We started with two overlapping cylinders (distance = 0 μm) describing the stellate cell morphology. Then we progressively increased their “pyramidalness”, i.e., the distance between the two dendritic bushes and generated a new interneuron population for each cylinder distance (Fig 8A; see Methods for details). The generated morphologies ranged from pure stellate cells (the interneuron used in the reference case), to cells corresponding to layer 2/3 pyramidal cells where the two cylinders were juxtaposed (the pyramidal neuron used in the reference case), to cells where the two areas were parted by several hundred micrometers (as in layer 5 pyramidal neurons). In all cases GABA synapses were distributed only on dendrites located inside the lower cylinder, while AMPA synapses were distributed over the entire dendritic tree (Fig 1B).

We found that the LFP signal from the 1000 interneurons was very weak for cylinder distances less than about 100 μm, corresponding to a 40% overlap between the two cylinders (see Fig 8B and 8C). The amplitude of the LFP signal increased with the cylinder distance together with the current dipole moment (Fig 8C and 8D; see Methods). The “transition distance” of about 100 μm is seen to be associated with the appearance of an inversion point in the LFP (Fig 8C) and with the establishment of a sizable dipole moment (Fig 8D). Above this transition distance the LFP became larger with larger cylinder separations, yet saturating somewhat for distances above about 250 μm, corresponding to our reference model of layer 2/3 pyramidal cell. This demonstrates that the lack of a sizable contribution to the overall LFP from our interneurons in the reference model was due to their stellate morphologies.

Below the inter-cylinder transition distance all proxies performed poorly with average fraction of variance explained across depths smaller than 70% (for 100 μm the range was <R^2^> between 0.37 and 0.64), but <R^2^> quickly saturated as soon as the dipole appeared (Fig 8E). <R^2^> was smaller for all proxies compared to the reference case (since the noise was larger due to the smaller number of neurons, i.e., 1000 neurons versus 4000 neurons for the reference case), but the ranking of performances for different proxies remained roughly the same: above the transition distance the fraction of variance explained by WS was 83%, RWS and the sum of absolute values of currents both explained 80%, the membrane potential and sum of synaptic currents 59%, while firing rate explained only 47% of the variance. Note that for inter-cylinder distances above the transition distance, the stable performance of the proxies were accompanied by stable values of the optimal coefficient α (Fig 8F). This result implies that the RWS we have found for populations of layer-2/3-like pyramidal cells, likely also can be applied to pyramidal cell populations with different morphologies, as long as they produce a dipolar LFP.

In order to verify that the assumptions we made to algorithmically construct the neuronal morphologies in the 3D network did not bias the results, we also did simulations using realistic morphologies obtained from anatomical reconstructions (see Methods). The spatiotemporal dynamics of these LFP signals was found to be qualitatively very similar to the one previously shown, and the agreement with proxies was even higher, with RWS reaching R^2^ = 0.95 (S4 Fig). This result indicates that our conclusions are not strongly dependent on the detailed branching patterns within the basal and apical dendritic bushes.

### Dependency of the LFP signal on the distribution of synapses

**Fig 9:**
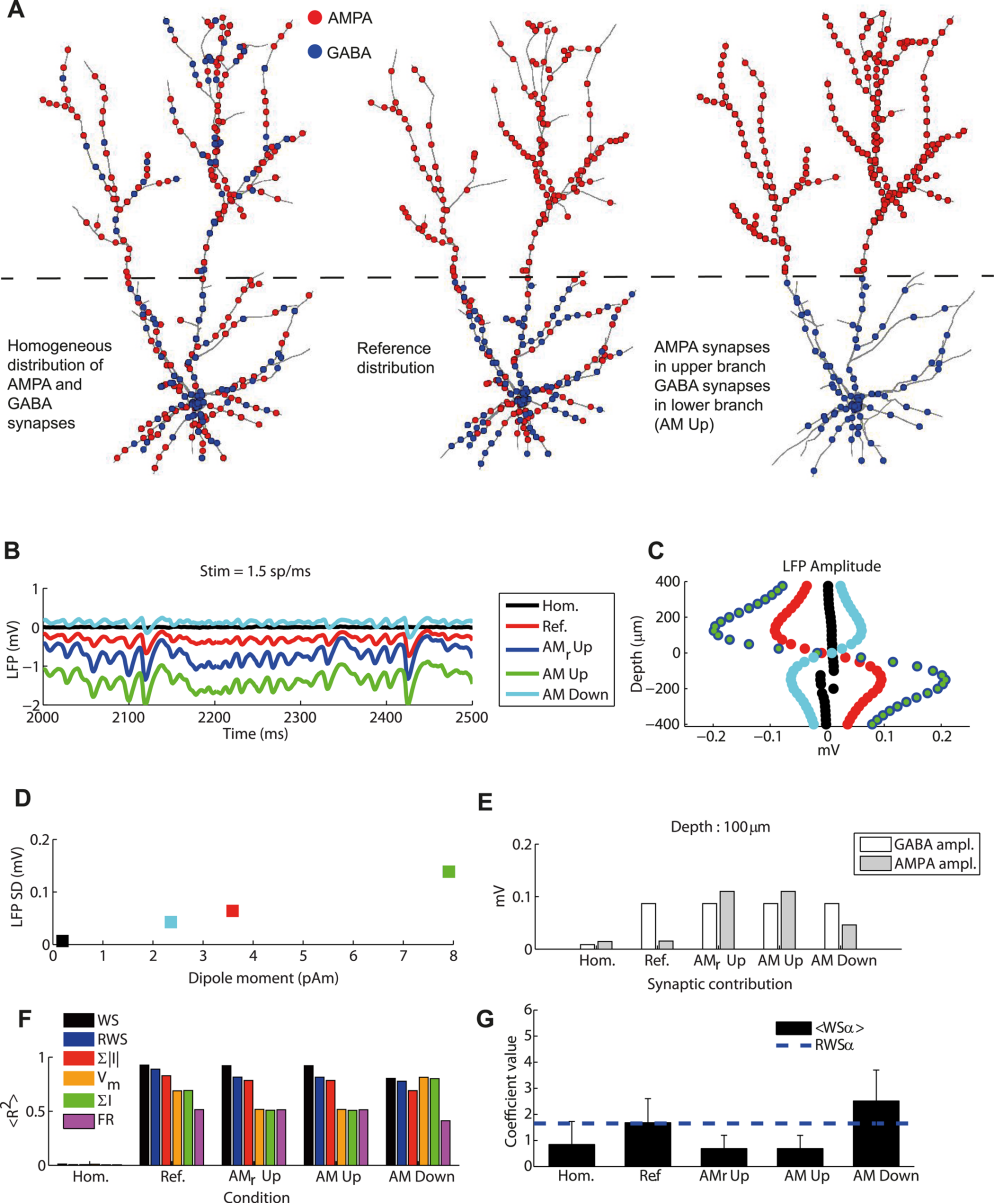
LFP signal and synaptic distribution. (A) Example cases for different synaptic distributions. Left: both AMPA and GABA synapse distributed over the entire surface of the cell. Center: GABA synapses distributed only in the lower cylinder, with AMPA synapses distributed over the entire cell. Right: GABA synapses distributed only in the lower cylinder and AMPA synapses only in the upper cylinder. (B) LFP time course for different synaptic distributions: the three configurations presented in (A) correspond to black, red and green lines, respectively. Additional configurations were tested where GABA synapses were located in the lower cylinder, thalamic synapses were in both cylinders and the cortical AMPA synapses were only in the upper cylinder (blue line, AM_r_ Up), and where all the AMPA synapses were located in the lower cylinder (cyan line, AM Down). (C) LFP amplitude as a function of depth (similar to Fig 2A) for different synaptic distributions. Blue and green markers were superimposed, illustrating that changing the position of the thalamic synapses does not alter the amplitude of the LFP (only but its mean value, cf. panel B). (D) Average LFP absolute amplitude over depths versus dipole moment (standard deviation over time) for the different synaptic distributions. (E) Contribution of AMPA and GABA currents to LFP fluctuation amplitudes for different synaptic distributions. (F) Average fraction of LFP variance explained by different proxies for different cylinder distances. Error bars are not displayed since they would not be visible in the figure. Same proxy arrangement as Fig 5C. (G) Mean and standard deviation across depths of optimal coefficients of α in the WS proxy as a function of synaptic distribution. Dashed line indicates the fixed coefficient of the RWS proxy. Note that since for homogeneous synaptic distribution the performance of the fit was low (see panel (F)), the fitted value of the relative weight of AMPA and GABA currents in contributing to the LFP signal has little significance.

In the reference case (Fig 1B) GABA synapses were distributed only in the lower cylinder while AMPA synapses were distributed homogeneously across all dendrites. In order to test how our results depended on this distribution we therefore evaluated all LFP proxies for a variety of synaptic distribution patterns. Fig 9A illustrates the three main different synaptic distributions tested: (1) a case where all synapses were distributed homogeneously, (Hom.) (2) the reference case (Ref.), and (3) a case where AMPA synapses were located only in the upper cylinder (AM Up), leading to a complete separation between AMPA and GABA synapses. We further considered two conditions where (4) AMPA synapses were located only in the lower bush leaving the upper bush empty (AM down) and where (5) AMPA cortical synapses were located in the upper bush while thalamic AMPA inputs were distributed homogeneously (AM_r_ Up).

Even though the parameters in the LIF network and thus the output activity remained precisely the same as before in these different situations, the corresponding LFP signal was dramatically altered by the choices of synaptic distributions (Fig 9B). The amplitude of the fluctuations was strongly affected, while the spatiotemporal features were only moderately altered. Note, however, that the position of the thalamic synapses only marginally affected the LFP fluctuations, and only the mean value of the LFP was affected. As a rule of thumb, we found that the more spatially segregated AMPA and GABA synapses are, the larger are the LFP fluctuations (Fig 9C). We further observed that the variation of the LFP amplitude on the synaptic distribution directly reflected changes in the magnitude of the current dipole moment (Fig 9D).

The individual contributions to the LFP from AMPA and GABA synapses were strongly dependent on the spatial distributions (Fig 9E): when synapses were distributed homogeneously, the contribution of their currents to the LFP signal was small as compared to when the synapses were segregated. Moreover, the AMPA contribution was larger when synapses were confined to the upper than to the lower cylinder. When the synapses were distributed homogeneously, the LFP signal was very weak resulting in poor performances for all LFP proxies (Fig 9F). When the cortical AMPA synapses were confined to the upper bush, the performance of the WS proxy was not affected, but a small decrease of 0.07 in the <R^2^> value was observed for both RWS and the sum of the absolute values of synaptic currents. For the same situation there was a larger decrease of 0.17 in the <R^2^> value to a global value of only 0.51 for both the membrane potential and the sum of synaptic currents. However, in the configuration in which AMPA synapses were confined to the lower bush and the LFP amplitude was small, the <R^2^> for the membrane potential and the sum of synaptic currents rose to 0.81 and 0.79 respectively, a value comparable to results for the WS and RWS proxies (0.80 and 0.78). This suggests that the advantage of using the WS and RWS proxies over, e.g., a membrane-potential proxy is particularly large when the AMPA and GABA synapses are spatially separated so that a large current dipole moment and a large amplitude LFP is generated (Figs 8C and 9D).

The best coefficients for WS strongly depended on the synaptic distribution (Fig 9G): When AMPA synapses were confined to the upper cylinder forming a strong current dipole moment, the optimal AMPA coefficients became larger than the GABA ones. Therefore, although the R^2^ value of RWS was still 0.82 under these conditions, a better result could be achieved with a proper tuning of the coefficients.

### Difference between current-based and conductance-based synapses for the LFP signal

To keep the consistency with the LIF network in which the synapses were current-based (see Methods), all LFP simulations considered until now were done using current-based synapses in the 3D network. However, some neuronal features may be better approximated by conductance-based synaptic models in which the postsynaptic currents (PSCs) depend on the local membrane potential and do not have a fixed shape as in the case of current-based synapses. To test this situation, we repeated our simulations by introducing conductance-based synapses in the 3D network. Synaptic time constants were left unchanged, while the peak conductance values were scaled to obtain PSC amplitudes equivalent to current-based synapses for the reference stimulus intensity 1.5 spikes/ms [47].

**Fig 10:**
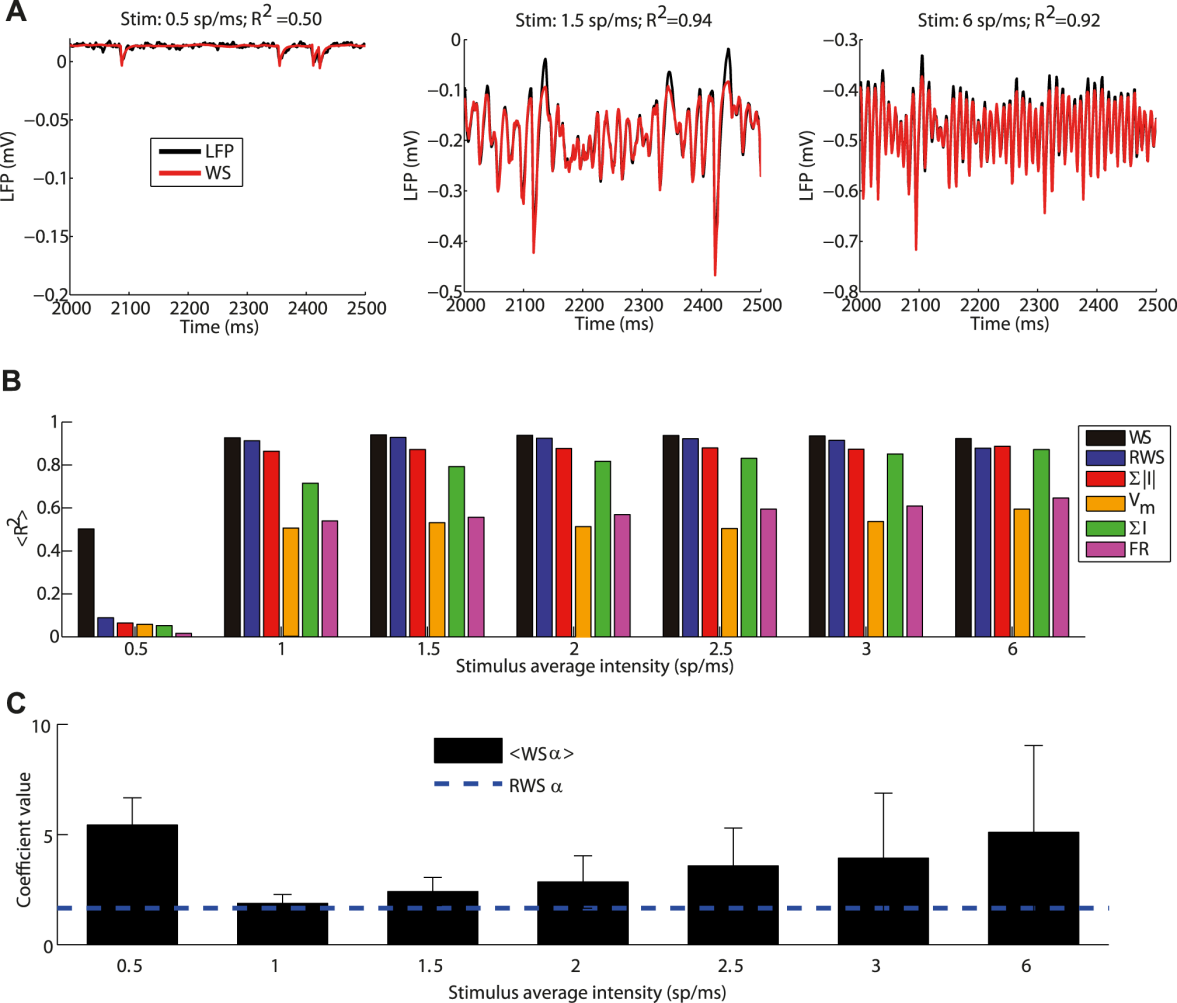
Effects of modulation of inputs with conductance-based synaptic model. (A) LFP (black line) for 150 μm depth position when the stimulation frequency was 0.5 spikes/ms (left), 1.5 spikes/ms (middle), 6 spikes/ms (right) and corresponding best fit with WS proxy (red). The depth was the one for which WS proxy performance was highest. The titles indicate the fraction of variance explained. Note the different vertical scales. (B) Average fraction of LFP variance over all depths explained by different proxies for different thalamic inputs. Error bars are not displayed since they would not be visible in the figure. Same proxy arrangement as in Fig 5C. (C) Mean and standard deviation across depths of optimal coefficients of α in the WS proxy as a function of thalamic input. Dashed line indicates the fixed coefficient of the RWS proxy.

While the simulated LFP amplitude was smaller when using conductance-based instead of current-based synapses (compare the three panels in Fig 10A with the three panels in Fig 6B and note the different y-axis scales), the time course was similar. We found that the explanatory power of the proxies was similar or better in all cases compared to the situation with LFPs computed with current-based synapses (Fig 10A): the R^2^ values for the RWS were in the range 0.91–0.93 for inputs between 1 and 3 spikes/ms, and 0.88 for 6 spikes/ms. We hypothesize that the main reason for the increase in performance was that the LFP contributions from different neurons were more correlated when synapses were conductance-based [47]. Note that in the case with conductance-based synapses, the performance of the membrane potential proxy is in the very low 0.5–0.6 range for R^2^ for all stimuli above 1 spikes/ms. This can be understood given that the membrane potential no longer depends linearly on synaptic input currents as in the case with current-based synapses.

The WS proxy coefficients for 1 spikes/ms inputs were rather similar to the current-based case, but when the input frequency was increased, the optimized value of the coefficient α, describing the ration of GABA to AMPA currents in the WS proxy, increased (Fig 10C). This likely reflects that for stronger stimuli the neurons were more depolarized, so that the average membrane potential was closer to the AMPA reversal potential and further away from GABA reversal potential. Consequently, the GABA versus AMPA PSC-amplitude ratio increased. Nevertheless, the RWS still performed well for all inputs (Fig 10B).

## Discussion

The main aim of this work was to develop an accurate, robust and an easy-to-use method to synthesize the LFP signal from output from a model network of LIF neurons. We simulated a biophysically realistic LFP signal using a population of morphologically detailed multi-compartmental neuron models and compared this LFP signal with several LFP proxy candidates extracted from the LIF network simulations. We found that a linear combination of summed and time-shifted AMPA and GABA currents in the LIF network explained a large fraction of the variance of the LFP of the 3D morphologically accurate network in nearly all conditions considered. Specifically, we identified a specific set of parameters (Eq 4), the so-called reference weighted sum LFP proxy (RWS), which accurately predicted the LFP time course for all considered electrode positions (Fig 5), and across different dynamic network states (Fig 6).

The fraction of LFP variance explained by the RWS proxy was only moderately affected by changes in neural morphology (Fig 8, S4 Fig), in synaptic distribution (Fig 9), or in synaptic dynamics (Fig 10). This LFP proxy was found to be very accurate for every condition considered in which the dipole generated by the synaptic currents was sizable and hence the amplitude of the LFP substantial. This LFP proxy only performed poorly in situations where the amplitude of the LFP signal itself was very small, i.e., at the inversion point or when the resulting current dipole moments from synaptic activation are small (homogeneous synaptic distributions (Fig 9), low activity (Fig 6)). Furthermore, we showed that despite the complexity of our LFP simulation setup (with 5000 different morphologies with realistic dendritic structures) the temporal evolution of the LFP was well captured by the RWS proxy based on synaptic currents with adjustment of only three parameters, the relative weight of the contributions from the two synaptic currents and the two synaptic delays. Our results further suggested that the RWS proxy can be used for a wide range of LIF network models and pyramidal-neuron morphologies to synthesize biophysically plausible LFP signals that can be compared with experimental LFP recordings. Table 5 describes how to properly use the proxy in a variety of conditions and modeling approaches.

**Table 5:** Summary of results for proxies and suggested adaption of results to other situations.

**REFERENCE TABLE FOR LFP PROXY USE**	
For the following reference conditions:	
• Layer 2/3 pyramidal cell (two neighboring dendritic bushes, (Fig 1B, left)	
• Synaptic distribution with GABA synapses in lower (basal) bush and AMPA synapses in both (basal+apical) bushes (Fig 9A, central panel)	
• Current-based synapses	
• 1.5 spikes/ms input	
• Electrode recording from the center of the 3D network at depth = 100 μm relative to inversion point	
**The optimal proxy (RWS)** is	
$LFP_{RWS}\left(r,d,t\right)=f_{RWS}\left(r,d\right)*Norm\left[\sum_{pyr}AMPA\left(t-6\;ms\right)-1.65\left(\sum_{pyr}GABA\left(t\right)\right)\right]$	
The proxy is, however, robust or easily adaptable to a variety of conditions as described below:	
**Aim**	**Action required**
Use a simpler proxy	Model temporal part of LFP as sum of AMPA and GABA currents or simply as GABA currents (Fig 4F and 4G)
Consider a different recording depth	Use the RWS (Fig 5C) unless you are within 50 μm from the inversion point, in which the LFP largely cancels out (Fig 2A and 2B)
Simulate LFP recorded with electrode radially displaced from the center of the 3D network	Use the RWS and determine amplitude f_RWS_(r,d) by means of Fig 2B
Vary rate of external synaptic input (input intensity)	Use the RWS (as long as the synaptic input is sufficiently strong to generate a sizable LFP, cf. Fig 6C)
Include/remove LFP contribution from transmembrane currents of stellate interneurons	Use the same RWS, since interneurons have a negligible effect on the LFP (Fig 3)
Simulate neurons with morphologies different from pyramidal cell of layer 2/3	Use the RWS for all morphologies in which two dendritic bushes are vertically more distant than 150 μm (i.e., for all cells except stellate cells that do not contribute to LFP), see Fig 8E.
Simulate neurons with synaptic distributions different from our reference case	As shown in Fig 9F and 9G:
	• Both synapses in both bushes: no appreciable LFP and no good proxy available
	• AMPA synapses only in upper bush: RWS performs well (R^2^ = 0.81), but to get better results increase relative weight of AMPA currents as follows:
	*LFP*_*RWS*_(*r*, *d*, *t*) = *f*_*RWS*_(*r*, *d*)
	$*Norm\left[\sum_{pyr}AMPA\left(t-6\;ms\right)-0.69\left(\sum_{pyr}GABA\left(t\right)\right)\right]$
	• AMPA synapses only in lower bush: RWS performs well (R^2^ = 0.77)
Simulate neurons with conductance synapses	Change AMPA and GABA coefficients as a function of input rate as indicated in Fig 10C

Thanks to its robustness, our proxy can expectedly be applied to models of any brain area in which the LFP is likely to be generated by one dominant population (as in the hippocampus with a single population of pyramidal cells). When there are two or more populations giving a significant contribution to the LFP (as is likely in whole cortical column model taking into account several pyramidal neuron populations, e.g., layer 2/3, layer 5, layer 6), the total LFP can be approximated as a suitable linear combination of individual contributions if information on the depth positions of the populations relative to the recording electrode is available. Comparison of the model LFP with experimental results might then be used to estimate the relative weights of the LFP contributions from the different populations.

### Comparison of candidate proxies

A major difference between the accurate LFP proxies using synaptic currents (sum of currents, WS, RWS) compared to the less accurate proxy based on firing rates is that a spike is a very local event in time, while the postsynaptic current following after a spike (as well as the contribution to the LFP) lasts for many milliseconds. So an instantaneous firing rate proxy like the ones we are considering based on firing rates cannot be expected to perform well (even with a fixed delay). In laminar population analysis (LPA, [30]) the LFP time course was rather assumed to be given by the measured firing rates convolved with a suitable (i.e., delayed exponential) kernel, the rationale being that spikes causally drive synaptic currents which in turn set up the LFP. The present RWS proxy is similarly constructed, effectively corresponding to a suitable weighted sum of exponentially convolved presynaptic spike rates corresponding to excitatory and inhibitory synaptic currents. The postsynaptic soma membrane potentials following presynaptic spiking is more low-pass filtered than the synaptic currents (and also the transmembrane return currents in the case of multicompartmental models) [29], and LFP proxies based on this dynamical variable will generally fail to predict the most rapid temporal changes in the LFP.

### Spectral properties

An interesting result is that all the proxies tested here displayed largely the same modulation of the LFP gamma power as a function of input intensity, both in terms of relative power modulation and peak frequency (Fig 7D and 7E). This is encouraging since we did not specifically aim to find a good prediction of the power spectrum when constructing the LFP proxies and estimating their parameters. We note however that no proxy is fully able to account for the low-frequency end of the spectrum (Fig 7A and 7B), which is overestimated by the membrane potential proxy and underestimated by the other proxies. If one is interested in a highly detailed reproduction of the whole LFP spectrum, preliminary results hint to the possibility of designing a WS fit optimized to match the spectrum instead than the spatiotemporal features and to define an LFP proxy that slightly differs from the RWS discussed above. However, the fraction of spectral variance explained by the RWS is already 0.91 (average over all stimulus intensities above 0.5 spikes/ms, standard morphology and synaptic condition) which likely is sufficient for most purposes.

### Model assumptions and limitations

In the present work we have focused on how the relationship between LIF variables and ground-truth LFP change when the 3D model features change, keeping the LIF model fixed. While different LIF networks would generate different activity and hence different synaptic currents, we expect roughly the same relationship between these synaptic currents and the generated LFP. Therefore, for any LIF network generating enough correlated activity to result in a sizeable LFP, we expect RWS to be a good proxy.

Our strategy had the advantage that we could vary the assumptions in the LFP-generating model, e.g., the distribution of synapses or neuronal morphologies, without affecting the spiking dynamics. The disadvantage of this approach is, however, that the 3D network does not match the LIF network in every respect; for instance, even though the synaptic input currents were identical in the two models, the resulting soma potentials in the multi-compartmental neurons were not identical to those in the LIF neurons (due to passive dendritic filtering). It is, however, unlikely that imposing identical somatic potentials, or identical currents entering the soma, in the two models would result in a more realistic LFP since large synaptic currents would be needed to overcome the passive filtering for distant synapses. Instead one could consider changing the synaptic weight distribution in the LIF network simulation to make the two models match better. Our focus here was to use LIF models as commonly used in the literature (typically using homogeneous weight distributions), but it would be an interesting topic for future studies to extract effective point-neuron synaptic weight distributions from the multi-compartmental population and use these in the LIF network simulations in order to make the two simulation environments even more similar.

We did not test different LIF network architectures or sizes, but we expect the RWS proxy to be applicable as long as the network displays a sufficient level of correlation. We have found in previous modeling studies [20,21] that correlated synaptic activity is necessary to create a sizable LFP signal, and in this case all cells in the dominant LFP-generating population will contribute. Making the network size larger or altering its connectivity would therefore likely not qualitatively change the form of the best LFP proxy (as long as a sufficient level of spiking correlations is maintained in the network). The LFP generated by larger populations, however, should be tested in further studies taking into account the summed effect of several cortical populations, across layers as well as heterogeneous spatial structure in the horizontal direction.

A limitation of the presented simulation setup is that it models only AMPA and GABA synapse contributions. However, most of our results pertaining to the proxy do not depend on the particular feature of the synapses and are therefore likely to extend to different synapses as well. For instance, it should not matter for the quality of our suggested proxy whether or not the synaptic weights are changing due to plasticity since the weight changes will be reflected in the synaptic currents extracted from the LIF network as well. Including slower synapses, such as NMDA synapses in the model setup, will on the other hand affect the LFP frequency content, particularly at low frequencies. This effect could be captured by a proxy including NMDA in the sum of synaptic currents with a weight depending on the number and spatial distribution of NMDA synapses. As with the synaptic weight distributions discussed above, the inclusion of NMDA synapses when computing the LFP proxy presupposes that it is also included in the LIF network model (which was beyond the scope of this study). Moreover, we did not model subthreshold active dendritic conductance [55], nor the active channels behind spike generation. The contributions from the latter is expectedly negligible for at least the low frequencies of the LFP [56] (but see [57–59]), while the effect of the former should be explored in future projects.

The present suggested proxy assumes the LFP contribution following spikes to be spatiotemporally separable, i.e., factorizable into a product of a function of time with a function of space [30]. Due to, for example, the intrinsic filtering effect [29,36,60] this is not strictly true as the spatial distribution of the transmembrane currents setting up the LFP depends to some extent on the frequency. However, if warranted the present proxy can be extended, for example by assuming a more detailed proxy consisting of a sum of such spatiotemporally separable kernels.

### Importance of this work for analytical studies of LFPs

Recently, we presented an analytical method to estimate the LFP spectrum from the dynamics of a LIF network [61] using as LFP proxy the sum of the absolute values of synaptic currents. By fitting a recurrent excitatory-inhibitory LIF network model to LFP recordings from monkeys presented with visual stimuli, we were able to estimate the LIF model that best fitted the observed LFP, and to predict at least in part the observed firing rate and some of the visual features in the receptive field that elicited the observed neural activity. In this recent work [61], the time evolution of the LFP was computed analytically from the LIF network as a function of the external input by applying linear response theory to the mean-field approximations of each kind of synaptic current separately and then summing their absolute values over pyramidal neurons (as in [15] and in Eq 2). In principle, it is possible to extend this analytical calculation by using the more efficient proxy presented here by simply changing the coefficients in the final sum of the synaptic currents. This paves the work for obtaining realistic analytical estimations of LFPs from recurrent LIF networks. As discussed in [35], an efficient analytical approach could be at the heart of the development of model-based analysis methods for performing inferential statistics of network models on LFPs, analogous to the role played by Dynamic Causal Modelling [62,63] in the analysis of EEG and fMRI recordings.

### Outlook

Here we studied proxies for the LFP produced by a local 3D network, corresponding to a single cortical layer. Experimentally recorded LFPs, however, are most likely containing contributions from several layers [20]. Therefore, a natural extension of this work would be to study the LFP generated by several connected 3D networks forming a full cortical columns [64,65] and determine how LFP proxies should be designed in this context. Since electrical potentials in the nervous tissue are assumed to add linearly, we expect LFP proxies to be constructed in largely the same manner as presented here, by summing synaptic contributions from different cortical layers, possibly with a weighting depending on the recording depths. Constructing the LFP signal from a full cortical column model [65] is the topic for a separate ongoing project [66].

We expect our proxy to also work well for other brain structures where pyramidal neurons are elongated and arranged in an almost parallel way, such as the CA1 and CA2 regions of the hippocampus. On the other hand, many subcortical structures have a neuronal architecture so different from the cortex that we that we cannot a priori expect the present rules of LFP prediction to be applicable. A possible future line of research will be to apply the combination of LIF dynamics and 3D morphology we used in this work to investigate such areas to find a compact way to study the mechanisms generating the LFP observed there.

We focused in the present study on the LFP signal, but finding good models for relating activity in spiking network models and experimentally measured signals is relevant also for other types of commonly recorded signals such as the EEG, MEG and VSD. Since the biophysical mechanisms generating these signals are in principle known, we believe our framework could be extended to also study other measurement modalities in the future.

## Methods

### Leaky integrate-and-fire (LIF) recurrent network model

We summarize here the structure of the LIF network that generated the spiking dynamics. We refer to [15,46] for full details. The network was composed of LIF neurons with current-based synapses whose time evolution was modeled as difference between exponentials (see below), fixed threshold, fixed refractory time [67], and fixed conduction delay of 1 ms. Subthreshold dynamics for each neuron were given by

$$\tau_{m}{\dot{V}}_{m}\left(t\right)=-V_{m}\left(t\right)+\sum \;PSC_{syn}\left(t\right)$$ (5)

where *τ*_*m*_ corresponded to the membrane time constant due to the leak, V_m_ was the membrane potential, and PSC were the occurring synaptic events as a function of time t. When the membrane potential V_m_ crossed a threshold value of 18 mV above resting potential, a spike occurred, the potential dropped to a reset value of 11 mV above the reset potential and no spike could be emitted for a refractory time of 2 ms.

Post-synaptic currents (PSCs) were determined by the spikes emitted by the pre-synaptic neurons in the LIF network as well as by the external inputs. The time course of PSCs was described by the difference of two exponentials simulating the opening and closing process of the synapse. The equation can be written with two first order differential equations introducing the auxiliary variable *x*:

$$\tau_{dsyn}\dot{PSC}\left(t\right)=-PSC\left(t\right)+x\left(t\right)$$ (6)

$$\tau_{rsyn}\dot{x}\left(t\right)=-x\left(t\right)+\tau_{m}\left(J_{syn}\;\sum_{syn}\delta (t-t_{syn}-\tau_{l})\right)$$ (7)

where *τ*_*r*/*dsyn*_ indicate the rise and decay times of the synapses, and *J*_*syn*_ indicates the synaptic strength. The latency time of the synapses *τ*_*l*_ was set to 1 ms. Compound synaptic currents were the linear sum of contributions induced by single pre-synaptic spikes occurring at time t_syn_. We included two types of synapses: AMPA and GABA. Pyramidal neurons had AMPA-like synapses, and interneurons had GABA-like synapses. Moreover, each neuron received excitatory external drive from (1) a long range cortico-cortical input activating AMPA synapses identical to those of the recurrent connections and (2) a thalamic input activating AMPA synapses with timescales and strengths resembling those of thalamocortical synapses. Synaptic parameters such as rise time, decay time, and amplitude depended on the type of synapse and the category of the post-synaptic neuron. All simulation parameters were in the range of the values reported in the literature [68–70] and are listed in Table 2. We verified that modifying these values did not affect the results qualitatively [15,46].

The default network was composed of 4000 pyramidal neurons and 1000 interneurons (Fig 1A). The LIF network connectivity was random and sparse, with a directed connection probability of 0.2 between any pair of cells. This resulted in an inhomogeneous connectivity with an average of 1000 pre- and post- synaptic connections for each cell. Each neuron received inhibitory and excitatory inputs from the neurons in the network, and also cortico-cortical and thalamic excitatory drives as described above. The long-range cortico-cortical drive represented the ongoing activity and the global contributions from other areas of cortex. Since ongoing cortical activity has most power for slow frequencies, this external drive was generated by an Ornstein-Uhlenbeck process with a low pass cut-off frequency of 10 Hz and a 0.25 mV standard deviation. Thalamic inputs were time-invariant in this set of simulations. Synapses carrying both types of external inputs were activated by random Poisson spike trains, with time-varying rates identical for all neurons. Details can be found in Table 1 and 2.

Simulations were computed with time steps of 0.05 ms and lasted 10.1 seconds, with the first 100 ms removed to limit the analysis to the network steady state.

Current based and conductance based LIF model source codes are identical to those used in [47] and are already available on the ModelDB sharing repository (http://senselab.med.yale.edu/ModelDB/ShowModel.asp?model=152539) with accession number 152539.

### Morphological model of a simplified layered cortical column

In order to compute the transmembrane currents that lead to an LFP signal, we constructed 3D morphological neuron models that captured the main morphological features of the cortical network described by point neurons in the LIF model. The algorithm used to construct the model morphologies was based on the fact that dendrites connect to their presynaptic partners in a manner minimizing their total length and conduction times from all synapses to the soma [71]. In such a framework, pyramidal cell dendrites can be seen as tree structures connecting as directly as possible to axons that are distributed in two distinct layers [51]. The generation of synthetic trees and subsequent analysis were performed using the TREES toolbox [71,72] [http://www.treestoolbox.org]. Two cylinders (250 μm radius and 250 μm height each) were therefore stacked to form a cylindrical column (Fig 1B). Somata of all cells were homogeneously distributed in the lower cylinder for both cell types. Axons were distributed isotropically within planes perpendicular to the cortical depth at random depth values. Pyramidal cells were connected first to the axons in the upper cylinder and then to the axons of the lower cylinder, this resulted in characteristic apical and basal dendritic trees. Stellate cells were only connected to the axons in the lower cylinder. Using 160 axons in each layer and a maximal reach distance of 150 μm for any dendrite to an input axon, resulted in realistic membrane surfaces, cable lengths and branch point number distributions (see S1 Fig). Diameter taper was selected to equalize synaptic democracy [73] and yielded good fits to the real counterparts with similar parameters for interneurons and pyramidal cells. The resulting pyramidal cell somatic input resistance was about 200 MΩ with specific membrane resistances R_m_ = 20000 Ωcm^2^ and axial resistances R_a_ = 150 Ωcm. The stellate cell input resistance was 175 MΩ with specific membrane resistances R_m_ = 10000 Ωcm^2^ and axial resistances R_a_ = 150 Ωcm. In order to spatially embed the simplified LIF network model, 4000 such pyramidal cells and 1000 interneurons were generated to populate the simplified columnar architecture. The resulting morphologies were then exported to NEURON [74,75] using the TREES toolbox functions.

In order to continuously alter the “pyramidalness” of cortical neurons as in Fig 8 we simply modulated the distance between the two cylinders corresponding to the two layers. With a distance of 0 μm, a perfect overlap of both cylinders, the resulting shape was symmetric as for the stellate cell. As the distance was increased between the two cylinders, the shape of the cortical cell traversed the shape of layer 2/3 pyramidal cells (distance of 250 μm), layer 4 pyramidal cells (distance of 350 μm) and layer 5 pyramidal cells (distance larger than 500 μm). The corresponding validation of morphological features compared with real dendrite reconstructions as can be observed in S1 Fig.

As a control for use of algorithmically constructed morphologies we derived an alternative model using multiple copies of real reconstructions distributed within the columnar arrangement. We used reconstructions from NeuroMorpho.org [76], made available by the group of Markram [76], of both stellate cells and layer 2/3 pyramidal cells in young rat somatosensory cortex. Since only 4 stellate cells and 36 layer 2/3 pyramidal cell morphologies were available, we reached the number of 1000 interneurons and 4000 pyramidal neurons by randomly selecting copies of the smaller sample and distributing them within the simplified columnar geometry. Cell body locations were chosen to preserve a fairly homogeneous distribution of membrane throughout the cylinders. This alternative model was then injected with the same synaptic current stimuli as the original model based on algorithmically developed morphologies, and yielded similar results (compare Fig 6 and S4 Fig).

### Connecting two simulation frameworks

Spike trains generated by the LIF network were used as input in the 3D network model used for LFP generation. Each multi-compartmental neuron model in the 3D network was associated with a given point neuron in the LIF network. To make sure the total synaptic currents in each cell were identical in the two simulation environments, we used the connectivity structure of the LIF network to determine the presynaptic LIF neurons for each postsynaptic multi-compartmental neuron in the 3D network. We triggered the synaptic currents in the multi-compartmental neurons of the 3D network at the precise times given by the spike trains generated by the presynaptic cells during LIF network simulations. Note that we did not take into account synaptic latency time. In the 3D network we associated with each presynaptic cell a single specific synapse in the postsynaptic cell. Synaptic dynamics in the 3D network was identical to the one in the LIF network (Eqs 4 and 5). In addition we recreated the external inputs (“Thalamic” and “Cortical”, see Fig 1A) used in the LIF network simulations and injected the same patterns of external spike trains in specific AMPA synapses in the 3D network neurons.

Since the LIF neuron model used in the LIF network simulations lacked spatial structure, we needed to make additional assumptions regarding the synapse placement when simulating the multi-compartmental neurons in the 3D network. The cylinders that were used to create the morphologies of the multi-compartmental models (see above) were also used to broadly define the synaptic regions. Our default setting was to place GABA synapses only in the lower cylinder, while AMPA synapses were placed in both cylinders. We tested also other scenarios in the “Dependency of the LFP signal on the distribution of synapses” subsection of Results (Fig 9). We randomly chose the detailed spatial position on the dendritic structure for each synapse, with the probability for a section to be selected being proportional to its membrane area, such that the resulting synaptic density was homogeneous within the selected cylinder

### Calculation of local field potential (LFP) from the morphological 3D network

We calculated the model LFP signal from the transmembrane currents in the multi-compartmental neuron populations based on volume conduction theory and the line-source approximation implemented in the Python package LFPy (http://lfpy.github.io/) [34]. We first simulated transmembrane currents resulting from synaptic activity using the NEURON simulation environment [74,75] after which extracellular potentials were calculated as a weighted sum of those transmembrane currents [31,32,34]. The extracellular potentials were computed for 32 equispaced vertically aligned points in space (simulating a laminar multielectrode), set at 25 μm intervals along the central vertical axis of the 3D network cylinder (Fig 1B). For the analysis illustrated in the subsection “Spatial distribution of simulated LFP signal” the recording locations were set at different distances from the vertical axis of the 3D network cylinder. To directly match the LIF network simulations, morphological neurons used current synapses in the reference case, except in the simulation discussed in the subsection “Difference between current-based and conductance-based synapses for the LFP signal” were conductance synapses were adopted (Table 3E). The calculations of transmembrane currents in the morphological model were performed using passive neuron models with the parameters listed above (Table 4). Following volume conductor theory, the model neurons were assumed to be surrounded by an infinitely sized extracellular medium with conductivity assumed to be real, scalar (the same in all directions) and homogeneous (the same everywhere) with *σ* = 0.3 S/m [77]. For further discussion on these assumptions see [32].

The Python codes we used to generate LFP from artificial morphologies injected with LIF spike dynamics are available on the LFPy official site (http://lfpy.github.io/).

### LFP proxies for LIF networks

We tested several simple models to match the LFP simulation based on the different variables describing the activity in the LIF network: firing rate, membrane potential, AMPA and GABA synaptic currents. We considered variables computed over the set of all pyramidal neurons, of all interneurons or both populations. We considered proxies based on these variables and on the simple sum or the sum of absolute values of synaptic currents as in [15,46]. Then we considered linear combinations of synaptic currents with different time delays. We tested the accuracy of the proxy in describing the time evolution of the LFP given by the morphological model by using the mean of squared values of the correlation coefficient R (which is equivalent to the fraction of variance explained). The quality of the proxy was tested separately for each depth. We computed the cross-correlation between the simulated LFP signal and the corresponding proxy and we determined the delay as the lag of the cross-correlation peak (see Fig 4). For this delay we determined the best linear fit using the Matlab function *polyfit* for single regressors and the Matlab function *regress* for regressor combinations. We estimated the quality of the proxy as the squared correlation coefficient between the best fit and the LFP. The proxy for each depth is defined by the optimal delays and the coefficients of the different components for regressor combinations. To compare the performance of the different proxies taking into account the different number of free parameters between WS, RWS and all the other proxies, we used the Bayesian Information Criterion (BIC, [54,78])

BIC=−2l+Klogn (8)

where *l* is the optimized loglikelihood function, *K* the number of estimable parameters and *n* the sample size. Under the assumption of Gaussian noise, −2*l* can be approximated as $constant+n\;\text{log}\frac{RSS}{n}$ [79] where RSS is the sum of the residual squares, so the BIC criterion becomes

$$BIC=n\;\text{log}\frac{RSS}{n}+K\;\text{log}\;n$$ (9)

which is the criterion we adopted in the manuscript.

## Supporting Information

S1 Fig Calibration of morphology of multi-compartmental models. (A) Layer 2/3 pyramidal cells: Comparison of amount of cellular membrane surface between anatomically reconstructed cells (left) and synthetic morphologies (right). Results shown in units of μm^2^ per cell for 25 μm bins of cortical depth. Green lines indicate profiles of individual cells and black lines are average traces. (B) As in panel A, but for stellate cells.
(TIF)

S2 Fig Summary of all tested proxies in the reference case. (A) Fraction of LFP variance explained by the different proxies at different depths. FR: Firing rate; Vm: membrane potential; AMPA/GABA: input current values; ∑I: sum of the two input currents; ∑|I|: sum of the absolute values of the two input currents. Quantities with _exc/_inh subscript are computed only over the set of excitatory/inhibitory neurons, e.g., AMPA_exc is the average AMPA current input into excitatory neurons. Quantities with no subscript are computed over all neurons. (B) Optimal time lag for different depths for same proxies as previous panel. (C) Fraction of variance explained by combination of AMPA and GABA currents with same coefficients as Eq (4) and different delays. Optimal (and reference) combination is indicated by an X.
(EPS)

S3 Fig Bayesian information criterion. Same as panel 5C but showing for each proxy the BIC value (see Methods) instead of the fraction of variance explained.
(EPS)

S4 Fig Input modulation with real morphologies. Same as Fig 6B and 6D when using real reconstructed morphologies of cortical stellate and layer 2/3 pyramidal cells (see Methods).
(EPS)
