# Supplementary figures and images for: Computing the Local Field Potential (LFP) from Integrate-and-Fire Network Models
Source: PLoS Comput Biol. 2015 Dec 14;11(12):e1004584. doi: 10.1371/journal.pcbi.1004584 (PMC4682791; doi:10.1371/journal.pcbi.1004584)

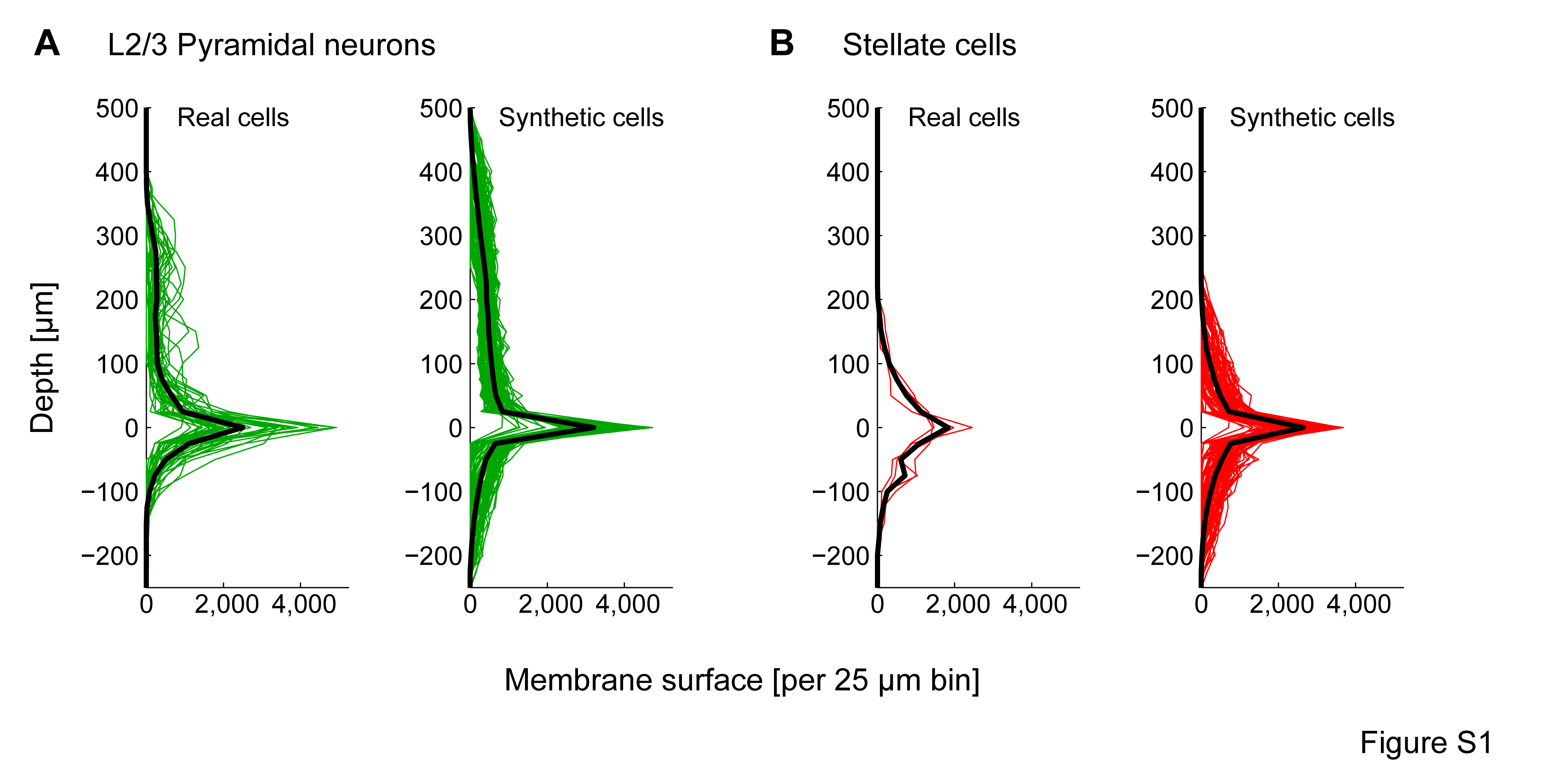

Supplement: S1 Fig — (A) Layer 2/3 pyramidal cells: Comparison of amount of cellular membrane surface between anatomically reconstructed cells (left) and synthetic morphologies (right). Results shown in units of μm2 per cell for 25 μm bins of cortical depth. Green lines indicate profiles of individual cells and black lines are average traces. (B) As in panel A, but for stellate cells. (TIF) [file pcbi.1004584.s001.tif]
